# Novel Urinary Biomarkers for the Detection of Bladder Cancer

**DOI:** 10.3390/cancers17081283

**Published:** 2025-04-10

**Authors:** Matthijs Oyaert, Charles Van Praet, Charlotte Delrue, Marijn M. Speeckaert

**Affiliations:** 1 Department of Clinical Biology, Ghent University Hospital, 9000 Ghent, Belgium; matthijs.oyaert@uzgent.be; 2 Department of Urology, Ghent University Hospital, 9000 Ghent, Belgium; charles.vanpraet@uzgent.be; 3 Department of Nephrology, Ghent University Hospital, 9000 Ghent, Belgium; charlotte.delrue@uzgent.be; 4 Research Foundation-Flanders (FWO), 1000 Brussels, Belgium

**Keywords:** bladder cancer, urinary biomarkers, molecular diagnostics, liquid biopsy, epigenetics, machine learning, multi-omics

## Abstract

Bladder cancer (BCa) is a highly recurrent malignancy that requires sensitive and noninvasive diagnostic and predictive markers. Conventional diagnostic tools, such as cystoscopy and urine cytology, are far from ideal in terms of sensitivity, specificity, and patient compliance. In this narrative review, the development of novel urinary markers for the diagnosis of BCa is highlighted, with a focus on their application in the clinical arena, detection accuracy, and future potential. An extensive analysis of new urinary biomarkers, including proteinuria-based tests, DNA methylation biomarkers, and RNA-based molecular panels, has been conducted. Various molecular tests, such as Cxbladder^®^, Bladder EpiCheck^®^, and UroSEEK, are highly sensitive and clinically valid. Urinary biomarkers provide a promising noninvasive alternative for traditional BCa diagnostics with enhanced specificity and the possibility of early diagnosis. Future research should focus on large-scale clinical validation and standardization of biomarkers to facilitate their use in routine clinical practice.

## Simple Summary

Bladder cancer (BCa) is a common disease that typically requires invasive methods, such as cystoscopy, for diagnosis and follow-up. However, these methods are painful, costly, and burdensome for patients. This review considers the newest noninvasive methods of BCa detection using urine-based biomarkers that can diagnose the disease with high sensitivity. These biomarkers include specific proteins, DNA changes, and RNA molecules that are correlated with BCa development. Urinary tests can offer an affordable and effective alternative to standard diagnostic methods and can guide new therapeutic strategies. Further proof is needed to confirm these biomarkers and advance their value in daily clinical routine, with the potential to improve early detection and treatment outcomes for patients with BCa.

## 1. Introduction

Bladder cancer (BCa) is a prevalent condition, with urothelial carcinoma (Uca) being the most common type, accounting for 95% of cases. The disease is classified as either non-muscle-invasive bladder cancer (NMIBC) or muscle-invasive bladder cancer (MIBC), with the latter being more aggressive and having a worse prognosis [1,2]. BCa is one of the top ten most often diagnosed malignancies worldwide [3]. The prevalence is especially high in industrialized places, such as North America and Western Europe, most likely due to environmental carcinogen exposure. Men are three to four times more likely than women to have the disease, and the risk increases significantly with age, with the majority of cases occurring in those over 65 years of age [4,5]. Tobacco smoke carcinogens are discharged in urine and directly damage the bladder epithelium, making it the major risk factor, accounting for 50–65% of all cases [4]. Other major risk factors include a history of recurrent urinary tract infections (UTIs), chronic inflammation, occupational exposure to aromatic amines and polycyclic hydrocarbons, and pelvic radiation therapy [6,7].

**Figure 1:**
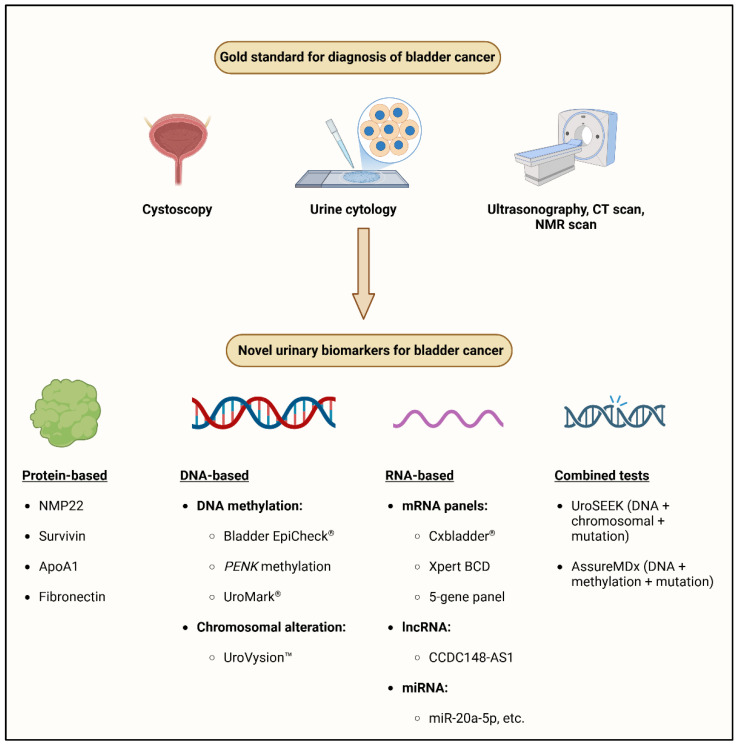
Overview of traditional and novel urinary biomarkers for bladder cancer diagnosis.

Given that NMIBC has a high recurrence incidence of 50–80% within five years, ongoing surveillance, early identification, and precise monitoring are critical for BCa [8]. Despite providing direct access to the bladder mucosa, cystoscopy, the current gold standard for diagnosis, is intrusive, costly, and uncomfortable for patients [9]. Urine cytology, a regularly used adjuvant, has high specificity (>90%) but low sensitivity (16–40%), especially for detecting low-grade malignancies [10,11]. Ultrasound, computed tomography (CT), urography, and magnetic resonance imaging (MRI) are complementary imaging modalities. However, they are insufficiently sensitive to detect early stage or recurring BCa (Figure 1) [12].

Due to the limitations of standard diagnostic approaches, reliable, noninvasive urine biomarkers that can aid in early detection, reduce the need for invasive procedures, and improve patient classification for surveillance and therapy are urgently needed. Over the last 10 years, improvements in molecular diagnostics have resulted in the discovery of promising urine biomarkers such as protein-based assays, RNA-based panels, and DNA methylation indicators. Urine-based diagnostics, such as Cxbladder^®^, Bladder EpiCheck^®^, UroVysion^TM^, and AssureMDx, offer early cancer detection and risk stratification while surpassing urine cytology in terms of sensitivity and specificity. Recent developments in computational oncology have emphasized the potential of integrating multi-omics data and machine-learning approaches to improve cancer diagnosis and prognosis [13]. This review investigated the development of urine biomarkers for BCa diagnosis, focusing on their clinical value, diagnostic accuracy, and future potential. We evaluated the newest existing biomarker-based diagnostics and identified potential molecular targets that could transform how BCa is diagnosed and treated in the future.

## 2. Traditional Approaches

### 2.1. Cystoscopy

Cystoscopy remains the gold standard for identifying BCa because it allows direct observation of the bladder mucosa and collection of biopsy samples for histological evaluation. However, it is an intrusive tool that causes patient discomfort, increases the risk of infection, and incurs significant expenses. Flexible cystoscopy has increased patient tolerance, but still requires specialized equipment and skilled workers. Cystoscopy has high sensitivity (81–97%) and specificity (77–87%) for detecting high-grade bladder tumors, but is less effective in identifying flat lesions such as carcinoma in situ (CIS), which may require additional fluorescence-guided cystoscopy for better detection [14].

Because of these limitations, urinary biomarker-based diagnostics, notably BCa screening, have been developed to supplement cystoscopy. Some assays, such as the Bladder EpiCheck^®^ and Cxbladder^®^, have shown promise in reducing the frequency of cystoscopic examinations in low-risk patients. However, they are not yet regarded as full substitutes for cystoscopy in standard clinical practice [15].

### 2.2. Urine Cytology

BCa is usually detected by hematuria, which is a common but ambiguous urine test. Blood in the urine can be produced by benign illnesses, such as kidney stones, trauma, or UTIs, but it can potentially indicate cancer. Microscopic hematuria is defined by the American Urological Association (AUA) as having at least three red blood cells (RBCs) per high-power field (HPF), or approximately 20–30 RBCs × 10^6^/L [16,17]. However, the incidence of BCa varies by hematuria type, with 4% of patients with microscopic hematuria and 16.5% of those with overt hematuria developing bladder cancer [18]. Despite being a common finding, hematuria has low specificity for BCa diagnosis and can result in wasteful diagnostic procedures [19]. Furthermore, hematuria may be intermittent, especially in early stage or low-grade malignancies, resulting in delayed or missed diagnosis. Peroxidase-based urine test strips may also produce false positives, resulting in excessive healthcare costs [20,21].

Urine cytology, which was first introduced by Papanicolaou and Marshall in 1945, remains the most reliable urine-based diagnostic method for detecting BCa. It is highly specific (95%), but has low sensitivity (16–40%), especially for low-grade cancers [22,23]. While urine cytology detects high-grade urothelial carcinoma (HGUC) with a sensitivity of 84%, its inability to detect low-grade cancers limits its use as a standalone diagnostic tool [10]. Despite being included in the AUA guidelines, only ~10% of patients with hematuria receive urine cytology, possibly owing to its inadequate sensitivity [17,18]. The Paris System for Reporting Urinary Cytology (TPS), introduced to standardize cytological interpretations, focuses on detecting HGUC based on specific morphological features [24,25]. Incorporating novel biomarkers, such as micronucleus (MN) and nuclear budding (NB) counts may further improve the diagnostic accuracy of urine cytology. A study of 117 urine cytology samples found considerably higher amounts of MN and NB in HGUC than in low-grade UC and benign cases (*p* < 0.001). The combination of MN and NB analyses improved the sensitivity (71.1%) and specificity (79.7%) for UC detection, suggesting possible prognostic importance [26].

In addition to cytology, urine particle analysis has emerged as an essential screening tool for clinical laboratories. Over the last 30 years, automated urine analyzers have increased standardization while reducing manual labor requirements [27]. Sysmex UF-5000 and UD-10 analyzers (Sysmex Corporation, Kobe, Japan) were evaluated for their capacity to detect abnormal urothelial cells in urine samples. In a 2021 investigation, automated analysis achieved a sensitivity of 59%, specificity of 82.1%, and 73% agreement with conventional cytopathology (Table 1) [28]. While these methods show potential, their sensitivity remains a limitation, especially for voided urine samples.

Overall, urine cytology is still an effective technique for detecting high-grade BCa. However, its limitations require the inclusion of other diagnostic tools, such as urinary biomarkers and imaging, to improve detection accuracy. Continued efforts to standardize interpretation using TPS and to incorporate automated cytological analysis may improve diagnostic precision in the future.

## 3. Urinary Biomarkers for Bladder Cancer Detection

### 3.1. Protein Biomarkers

Urinary protein indicators have gained popularity as noninvasive methods for the early identification and surveillance of BCa. As urine is in intimate contact with the bladder urothelium, it is an excellent medium for detecting cancer-specific proteins released by tumor cells. Although several protein-based assays have been developed and studied for their potential as diagnostic tools, their practical application is limited because of their unequal sensitivity and specificity [33].

Nuclear Matrix Protein 22 (NMP22), produced by apoptotic bladder cancer cells, is one of the most studied urine protein markers. NMP22 has Food and Drug Administration (FDA) approval for the detection of BCa because of its sensitivity of 55–65% and specificity of 70–85%. However, the possibility of false-positive results for hematuria or UTIs may limit its standalone use [34,35]. Survivin, an anti-apoptotic protein, has emerged as a promising biomarker [36]. Furthermore, apolipoprotein A1 (ApoA1), a protein involved in lipid metabolism, has shown diagnostic promise in distinguishing bladder cancer from other diseases. Transforming growth factor-beta (TGF-β) and fibronectin are associated with BCa progression. TGF-β governs tumor microenvironment remodeling and immune evasion, whereas fibronectin promotes epithelial–mesenchymal transition (EMT), leading to increased tumor invasiveness [37].

The current research trend is to combine many protein markers into multiplex panels to improve the overall sensitivity and specificity. Advances in proteomics and artificial intelligence (AI)-based biomarker profiling may increase the therapeutic efficacy of urine protein biomarkers in detecting BCa [38].

### 3.2. Molecular Biomarkers and Multi-Gene Panels

#### 3.2.1. Cxbladder^®^

Cxbladder^®^ assays are diagnostic urinary biomarker tests based on reverse transcription–quantitative PCR (RT–qPCR). They serve two primary purposes: (a) risk stratification for patients undergoing evaluation for hematuria (Cxbladder Detect and Cxbladder Triage) and (b) surveillance of patients previously treated for UCa (Cxbladder Monitor) [39,40,41]. These tests assess the messenger RNA (mRNA) expression of five biomarkers, including the inflammatory marker C-X-C Motif Chemokine Receptor 2 (CXCR2) and four genes linked to ulcerative colitis: cyclin-dependent kinase 1 (CDK1), midkine (MDK), insulin-like growth factor binding protein 5 (IGFBP5), and Homeobox A13 (HOXA13) [42]. The presence of CXCR2, which is strongly expressed in neutrophils, helps reduce interference from background inflammation, increasing the distinction between nonmalignant diseases and UCa [41]. Clinical trials on patients with microhematuria or extensive hematuria have shown that Cxbladder Detect has moderate sensitivity and high specificity, allowing excellent risk classification for UC [43,44,45]. Furthermore, the Cxbladder Triage has demonstrated good sensitivity and a strong negative predictive value (NPV), making it a useful rule-out test for UCa (test-negative tool) [43,44,45,46]. However, its lower specificity (46%) may increase false-positive rates, which should be considered in clinical decision making. These findings indicate that Cxbladder Detect and Triage may be a more reliable, noninvasive alternative to urine cytology in some patient populations, but more comparative research is needed to validate this advantage. In clinical practice, Cxbladder Detect can be used as an initial screening tool for patients with microhematuria, allowing those who receive negative results to avoid more invasive tests. Urine cytology can be performed on people who have positive results, increasing the positive predictive value of noninvasive assessment. Similarly, for individuals being monitored for recurrent UCa, Cxbladder Monitor has proven to have good sensitivity and NPV [43], allowing physicians to confidently rule out recurring disease in some circumstances and perhaps reduce the frequency of invasive procedures for some patients. However, there is a risk of false positives owing to its low specificity (39%), which may result in unnecessary follow-up testing.

**Figure 2:**
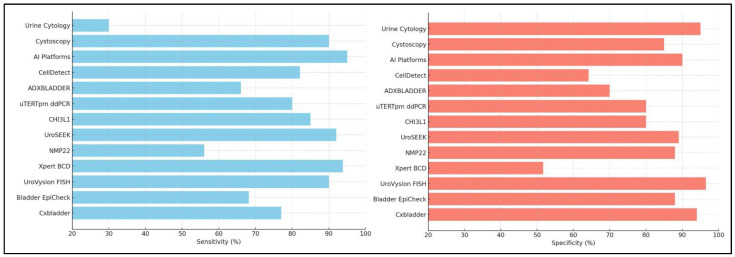
Comparison of sensitivity and specificity of diagnostic methods for bladder cancer detection.

Comparing Cxbladder to other urine biomarker tests such as UroVysion FISH and NMP22, can provide more information about its clinical usage in various scenarios. Cxbladder revealed analytical sensitivity ranging from 12.5 to 31.1 RNA copies/mL of urine for the CDK1, MDK, IGFBP5, and HOXA13 UC biomarkers, as well as 68.9 RNA copies/mL for the inflammatory biomarker CXCR2, fulfilling all pre-specified analytical performance criteria. The diagnostic performance of Cxbladder was strong, with Cxbladder Detect reaching 77% sensitivity, 94% specificity, 68% positive predictive value (PPV), and 96% NPV (Figure 2) [47]. Cxbladder demonstrated excellent repeatability (>85% concordance between laboratories) and analytical accuracy (≤10.63% error across all biomarkers) [45]. Despite these encouraging findings, further independent studies in larger and more diverse populations are required to determine whether they can be employed in a variety of therapeutic scenarios.

#### 3.2.2. Bladder EpiCheck^®^

Bladder EpiCheck^®^ (BE) is emerging as a potential urinary biomarker for the diagnosis and follow-up of NMIBC. Fifty patients diagnosed with NMIBC who were scheduled to undergo a second transurethral resection of the bladder tumor (TURBT) were prospectively included in the study. BE showed high specificity and NPV for identifying residual tumors, especially in low-risk NMIBC patients. In patients with initial tumor stage Ta, the specificity was 100% and the NPV was 87.5%, demonstrating that BE could confidently rule out residual disease. For the original tumor stage T1, the specificity was 71.4% and the NPV was 68.2%, indicating modest efficacy in detecting residual tumor presence. However, additional validation in larger cohorts is required. Similarly, in patients with high-grade malignancies, BE showed 77.8% specificity and 75% NPV. When all NMIBC patients were included, the specificity and NPV for predicting the remaining tumors at the second TUR were 78.6% and 73.3%, respectively. BE positivity rates were significantly higher in T1 (*p* < 0.037) and high-grade tumors (*p* < 0.002), suggesting that BE could serve as an independent predictor of residual tumor presence [48]. BE’s ability to detect high-risk cases supports its potential use in clinical decision making, particularly in determining the necessity for a second TUR. While these results are encouraging, it is crucial to compare BE diagnostic accuracy to other known urine biomarkers for NMIBC to contextualize its therapeutic usage. Furthermore, the study’s limited sample size (50 patients, 90% of whom were men) limits its generalizability, encouraging more diverse populations to be the focus of future research. One noteworthy finding of this study is that BE has the potential to reduce the number of unnecessary second TUR procedures, which are now routinely performed to ensure complete excision of high-risk NMIBC. The notion that reducing surgical procedures might reduce healthcare costs while improving patient quality of life requires more cost-effectiveness evaluations and long-term outcome research. BE’s noninvasive nature, high accuracy, and exact urine biomarkers may help patients receive better care, save money on medical bills, and live longer by preventing the need for further surgery when no residual tumor is found. Although BE, a highly specific and noninvasive urine biomarker, may enhance patient care, more research is required to ascertain its treatment effectiveness across different NMIBC risk categories [49].

#### 3.2.3. Xpert Bladder Cancer Detection

The Xpert Bladder Cancer Detection in Emergency Setting Assessment (XESA) Project was a prospective, single-center study that assessed the Xpert Bladder Cancer Detection (Xpert BCD) test as a BCa diagnostic tool in emergency departments. This study revealed that, although the evidence is still preliminary, Xpert BCD may be more accurate than urine cytology in terms of diagnosis, particularly for high-grade BCa. This study included 76 individuals with gross hematuria who visited the emergency room. Xpert BCD had 93.8% sensitivity, 51.7% specificity, 34.1% positive predictive value (PPV), and 96.9% negative predictive value (NPV) for detecting any BC. Xpert BCD demonstrated 100% sensitivity, 47.8% specificity, 20.5% PPV, and 100% NPV for high-risk malignancies (HG pTa- ≥ pT1 BC). These findings indicate that, while Xpert BCD has high sensitivity for detecting BCa, it has low specificity, meaning that some false positives occur. In comparison, urine cytology has 100% specificity but a far lower sensitivity of 25% for any BC and 44.4% for high-risk BC [50]. Although white-light cystoscopy (WLC) is incredibly accurate (100 percent sensitivity for both any and high-risk BCa), it is invasive and requires specialized tools and personnel. The area under the curve (AUC) for identifying any BCa was 0.73 for Xpert BCD, 0.62 for UCa, and 0.93 for WLC. Even if Xpert BCD is used as an additional non-invasive method rather than a replacement, WLC remains the gold standard based on statistically significant differences between the two treatments.

The study also examined the use of Xpert BCD as a screening tool in emergency rooms to prevent unnecessary WLC procedures. Using Xpert BCD as the first screening method may have prevented 42.1% (32 out of 76) of WLC procedures in this study, with only one case of low-grade BCa missed (6.3%). Larger, multicenter studies are needed to confirm these findings. In contrast, utilizing urinary cytology as a screening test might have prevented 94.7% (72/76) of WLC surgeries, while missing 75% (12/16) of BCa cases, including 66.7% (8/12) of high-risk malignancies. This shows that Xpert BCD might be used as a triage tool for patients with hematuria, although its clinical use has yet to be thoroughly proven.

According to the cost analysis, Xpert BCD can translate into cost savings by reducing resource utilization and preventing unnecessary urinary cytology and WLC therapy. The standard diagnostic process of urinary cytology and WLC costs every patient €370.86, whereas the application of Xpert BCD in guiding decision making costs €459.67 for positive results and €248.55 for negative results. The overall yearly cost savings were estimated at €64,260.72 in direct expenditures, increasing to €79,035 when healthcare resource optimization was included, on an estimated 400 eligible patients per year. However, these are only estimates based on healthcare system-specific factors such as false-positive rates and payment rules. The study has several limitations. The sample size was small (76 people were evaluated) and patients were difficult to enroll, especially in an emergency setting. Furthermore, catheter-induced urinary tract irritation may lower the specificity of false-positive results. 

Another primary issue was anticipatory positive (AP) outcomes. AP outcomes indicated that the test could detect early molecular changes since a number of those who tested Xpert BCD-positive but WLC-negative subsequently developed BCa later, although long-term studies are required to validate this perspective. Only WLC-positive patients underwent transurethral resection of bladder tumor (TURBT), resulting in partial verification bias, which might have overestimated the sensitivity and specificity. Furthermore, this study did not compare Xpert BCD with other developing urine biomarkers for BCa, such as UroVysion or CxBladder, which might provide further context for its clinical relevance. Future research should address these comparisons and evaluate Xpert BCD in larger, more diverse patient populations.

#### 3.2.4. Cytokeratin Fragment-19

Urinary cytokeratin fragment-19 (CYFRA21-1) is a protein biomarker. It is a soluble fragment of cytokeratin 19, an intermediate filament protein primarily expressed in epithelial cells. CYFRA21-1 is released into body fluids, including urine and blood, owing to cell turnover, apoptosis, or necrosis, particularly in cancerous tissues. In BCa, malignant urothelial cells release cytokeratin 19 fragments into the urine, making CYFRA21-1 a promising noninvasive biomarker for early identification, monitoring, and prognosis. However, CYFRA21-1 is not limited to BCa, as higher levels have been observed in different cancers (e.g., lung and gastrointestinal tumors), as well as non-malignant diseases such as UTIs and inflammation [51,52].

A single-center study [53] with a cross-sectional design included 154 adult patients aged ≥ 18 years who had hematuria and were suspected of having cancer based on imaging results. Urine samples were obtained prior to cystoscopy, and CYFRA21-1 concentrations were determined using enzyme-linked immunosorbent assay (ELISA). Among the total 154 individuals, 92 were diagnosed with BCa, and their urine CYFRA21-1 levels were considerably higher than those in the non-BCa group. The diagnostic performance of CYFRA21-1 was assessed using receiver operating characteristic (ROC) curve analysis, which yielded an AUC of 0.608, indicating only moderate diagnostic accuracy, which is lower than that of other established urinary biomarkers. A cut-off value of 13.3 ng/mL was established to optimize the diagnostic capacity. The test exhibited a sensitivity of 80.4%, which is acceptable, but falls short of the threshold generally expected for an excellent screening biomarker (>90%). However, its specificity was 43.5%, indicating that it was less successful in ruling out BCa in unaffected individuals and had a high false-positive rate, most likely due to non-malignant diseases affecting urinary CYFRA21-1 levels. The PPV was 67.9%, indicating that almost 68% of patients with a positive CYFRA21-1 result had cancer, while the NPV was 60%, indicating that 60% of patients with a negative result were accurately classified as cancer-free. The positive likelihood ratio (PLR) was measured at 1.425, suggesting that a positive test modestly increased the probability of having BCa but was not definitive on its own. While urinary CYFRA21-1 demonstrated moderate diagnostic accuracy, its low specificity limits its standalone clinical utility. Comparisons with other urinary biomarkers, such as UroVysion, NMP22, and Xpert BC, suggest that CYFRA21-1 may be less specific and therefore more prone to false positives [51,54]. Nevertheless, its moderate sensitivity makes it a potential adjunct to other diagnostic modalities rather than a replacement for standard tools. Instead, CYFRA21-1 could be integrated into a multimodal approach, complementing other diagnostic techniques such as cystoscopy, urine cytology, and additional biomarkers to enhance specificity and overall clinical utility. Given its non-invasive nature, CYFRA21-1 testing could help stratify patients who require further invasive procedures, potentially reducing unnecessary cystoscopies in low-risk individuals. However, its real-world clinical feasibility depends not only on its diagnostic accuracy but also on factors such as cost-effectiveness, accessibility, and test standardization (areas that require further investigation).

Further multicenter and longitudinal studies are needed to refine its clinical application, establish optimized cut-off values, and determine its role in BCa management. Additionally, cost-effectiveness analyses should be conducted to assess whether CYFRA21-1 testing offers tangible benefits over existing diagnostic pathways [53].

#### 3.2.5. Uromonitor and TERTpm ddPCR

The clinical utility of urinary telomerase reverse transcriptase (TERT)pm ddPCR as a promising noninvasive test for diagnosing primary BCa has been evaluated [55]. The study prospectively collected urine samples from 142 patients, including 74 with primary BCa, 20 with recurrent BCa, and 48 with benign urological conditions, all prior to surgical resection. These samples were analyzed using uTERTpm ddPCR, Uromonitor, and urine cytology to compare their diagnostic accuracies. The uTERTpm ddPCR test demonstrated the highest sensitivity (79.7%) for detecting primary BCa, which was significantly higher than those of urine cytology (59.5%, *p* = 0.005) and Uromonitor (56.8%, *p* = 0.001). However, the specificity values were not disclosed, making it unclear how well each test prevented false positives. Although the specificities of all three tests were statistically comparable, numerical specificity values should be provided for a comprehensive evaluation. The uTERTpm ddPCR test produced a better diagnostic yield, particularly in patients with primary BCa. Additional statistical analyses, such as effect size and confidence intervals, will validate this result. Stratified analysis revealed that the uTERTpm ddPCR test was extremely sensitive for the detection of high-grade NMIBC and muscle-invasive BCa; conventional cytology has low sensitivity for diagnosing both of these conditions. This moderate-to-high sensitivity is crucial for early identification and prompt intervention, thereby reducing the need for invasive diagnostic procedures, such as cystoscopy. 

Despite these positive findings, this study has some limitations that must be addressed. First, this was a single-center study with a limited sample size, which may restrict the generalizability of the results. Furthermore, selection bias may have influenced the reported diagnostic performance, particularly in the symptomatic group. Further validation in independent multicenter cohorts is required to corroborate these findings. Conversely, while urine cytology showed moderate diagnostic accuracy, its sensitivity was notably lower, particularly for detecting low-grade NMIBC, which is consistent with previous studies demonstrating its limitations as a standalone diagnostic tool. Additional statistical analyses, effect sizes, and confidence intervals may also confirm this finding. Stratified analysis demonstrated that the uTERTpm ddPCR test was extremely sensitive for the diagnosis of high-grade NMIBC and muscle-invasive bladder cancer, both of which were insensitive to routine cytological diagnosis.

In conclusion, uTERTpm ddPCR seems to be a promising noninvasive test for the diagnosis of primary BCa, especially in patients with muscle invasion and high-grade NMIBC. To demonstrate its therapeutic relevance and potential importance in reducing the need for cystoscopy, larger sample numbers, independent validation cohorts, and comparisons with other urine biomarkers are required [56].

#### 3.2.6. CellDetect Assay

The CellDetect test has been investigated as a possible noninvasive diagnostic tool for BCa, especially recurrent BCa, where it was found to be more sensitive and specific than standard urine cytology. In a retrospective analysis [57], 148 urine samples from patients with hematuria or irritative voiding symptoms were tested using the CellDetect test, which was confirmed by cystoscopy or TURBT. Patients were divided into two groups: Group P (84 initial bladder cancer cases) and Group R (64 recurrent bladder cancer cases). The study utilized descriptive statistics, but did not report confidence intervals, *p*-values, or ROC curve analysis, which would be necessary to determine statistical significance. Among the 148 patients, 115 were positive in the CellDetect assay (68 in Group P and 47 in Group R), and 134 were confirmed as malignant after pathological evaluation. The overall sensitivity and specificity of CellDetect for BCa diagnosis were 82.1% and 64.2%, respectively. When broken down by subgroup, Group P was 81.0% sensitive and 50.0% specific, whereas Group R was more sensitive, with 85.2% sensitivity and 83.3% specificity. These results suggest that CellDetect may be particularly valuable for diagnosing recurrent BCa if its specificity can be enhanced. However, without direct statistical comparisons (e.g., *p*-values or effect sizes), it is uncertain whether the distinctions between the main and recurring instances are statistically significant. While CellDetect appears to offer an improvement over traditional urine cytology, a direct statistical comparison with cytology’s performance in this study was not provided, making it difficult to quantify the actual degree of improvement. In addition, other established urine biomarkers widely utilized in clinical practice, including UroVysion, NMP22, and CxBladder, were not compared with CellDetect in the current study. These comparisons would provide a more complete appreciation of the comparative utility of CellDetect for bladder cancer diagnosis. 

This study had some limitations. Owing to the patients’ prior suspicion of BCa, selection bias may have been present in the retrospective design. Additionally, it is unknown whether CellDetect performs similarly in asymptomatic individuals or in a larger and more diverse population. This places its external validity in doubt. Future prospective multicenter studies with larger populations are necessary to validate these findings.

In conclusion, CellDetect is a promising noninvasive BCa diagnostic test, particularly for recurrent disease when specificity increases. Additional studies, such as large-scale statistical analysis, comparison with other urine biomarkers, and prospective cohort validation, are needed to establish its actual therapeutic application and possible contribution to the reduction of cystoscopy needs [58].

#### 3.2.7. UroVysion^TM^

UroVysion™ fluorescence in situ hybridization (U-FISH) is a widely used molecular cytogenetic assay to detect chromosomal abnormalities associated with BCa. U-FISH was approved by the FDA in 2001 for detecting BCa and in 2005 for post-surgical surveillance. The assay utilizes fluorescent DNA probes targeting chromosomes 3, 7, and 17, and the 9p21 locus (P16 tumor suppressor gene), which frequently exhibit aneuploidy or deletions in bladder cancer cells. These chromosomal aberrations are strongly associated with tumor aggressiveness and progression. According to several studies, U-FISH is more sensitive and specific than urine cytology, especially for the detection of high-grade bladder cancer. The 48% or so sensitivity of urine cytology is easily matched by the 80–100% sensitivity of U-FISH. This test also works well in the scenario of high clinical suspicion with negative or indeterminate cytology results. Moreover, hematuria and urinary tract infections, which are known to consistently disrupt cytological assays, cause fewer disruptions in U-FISH. Due to the high recurrence rate of BCa, ongoing monitoring is required. U-FISH is commonly used to detect residual disease and forecast tumor recurrence following TURBT. A study discovered that individuals with positive U-FISH findings after the first TURBT had a considerably greater recurrence rate than those who tested negative [59]. Positive U-FISH results following TURBT indicate the necessity for early second-look TURBT or enhanced intravenous treatment. U-FISH is an important tool for predicting treatment response in Bacillus Calmette–Guérin (BCG) patients. Several studies [60,61,62] have found that sustained U-FISH positivity 6 weeks or 3 months after BCG administration is significantly associated with an increased risk of recurrence and progression. Patients with a positive U-FISH test post-BCG are 3–5 times more likely to experience recurrence and 5–13 times more likely to have disease progression than those who test negative. Therefore, U-FISH can guide clinicians in adjusting the treatment strategies and follow-up intervals.

Several noninvasive urine biomarkers, including NMP22, Bladder EpiCheck^®^, and ImmunoCyt, have been developed to identify bladder cancer. However, U-FISH remains one of the most reliable. A comparative study revealed that U-FISH has the best sensitivity (77%) and specificity (98%), outperforming cytology and other biomarker tests. However, its low sensitivity for detecting low-grade BCa remains a disadvantage, as low-grade tumors may lack significant chromosomal alterations. One of the most significant advantages of U-FISH is its excellent sensitivity and specificity, particularly for high-grade bladder cancer. Compared with standard urine cytology, which frequently misses early malignant alterations, U-FISH provides a more accurate evaluation of chromosomal abnormalities associated with UCa. This makes it a useful tool for the early diagnosis of aggressive bladder cancer, when treatment is most effective. 

Another notable benefit of U-FISH is that it detects tumor recurrence earlier than cystoscopy does. Although cystoscopy is still the gold standard for bladder cancer monitoring, it is an intrusive and somewhat unpleasant technique that requires a direct view of the lesions. In contrast, U-FISH can detect chromosomal changes in urine samples prior to apparent tumor growth, allowing for early intervention and better patient outcomes. Moreover, U-FISH is less affected by inflammatory conditions, hematuria, and UTIs, which often leads to false-positive results in cytology-based tests. This makes it especially valuable for patients undergoing intravenous treatment, because inflammatory reactions may conceal cytological data. By reducing the influence of confounding variables, U-FISH improves the diagnostic accuracy and eliminates unnecessary procedures. U-FISH is also useful for evaluating treatment response, especially following transurethral resection of bladder tumor (TURBT) and Bacillus Calmette–Guérin (BCG) therapy. According to previous studies, sustained U-FISH positivity following BCG therapy is a significant predictor of recurrence and advancement, allowing doctors to adapt treatment strategies ahead of time. This makes it a significant resource in directing long-term patient care. 

Despite these benefits, U-FISH has certain drawbacks. One of its primary drawbacks is its low sensitivity to low-grade bladder cancer. As low-grade tumors often exhibit fewer chromosomal abnormalities, they may not be detected as reliably as high-grade malignancies. This limitation reduces the effectiveness of U-FISH in certain patient populations, necessitating complementary diagnostic approaches. The fact that U-FISH requires specific laboratory knowledge for the precise interpretation of results presents another difficulty. In contrast to traditional cytology, which is more accessible and simpler to perform, U-FISH requires skilled professionals for fluorescence microscopy and DNA probe hybridization. Accessibility may be restricted in situations with fewer resources or smaller medical institutions. Finally, one of the biggest obstacles to the broad adoption of U-FISH is its high cost. In terms of test execution and the necessary laboratory equipment, U-FISH is costlier than conventional urine cytology. This might restrict its application in situations where cost-effectiveness is a key consideration, especially when treating patients who need frequent follow-up [59].

#### 3.2.8. NMP22 BladderChek Test

The NMP22 BladderChek test is a non-invasive, high specificity, moderate sensitivity urine biomarker that is ideal for diagnosing high-grade BCa and monitoring recurrence. Its moderate sensitivity, however, precludes its usefulness as a standalone diagnostic test; it is best used in conjunction with standard diagnostic protocols. While it cannot replace cystoscopy or other gold-standard tests, it may improve bladder cancer detection and surveillance, particularly in high-risk populations and recurrent disease settings.

In an extensive systematic review and meta-analysis [34], which included 23 studies for qualitative assessment and 19 studies for quantitative synthesis, pooled data indicated that the NMP22 BladderChek test had a sensitivity of 56% and specificity of 88%. The diagnostic odds ratio (DOR) was 9.29, with an AUC of 0.83, indicating moderate-to-good diagnostic discrimination. Direct statistical comparisons (*p*-values) of significance between NMP22 and other urinary biomarkers to determine differences were not included. Performance variations based on tumor stage and grade were evident using this test. Extremely low sensitivity was encountered within early stages, with detection frequencies of 13.68%, 29.49%, and 34.62% reported for Ta, T1, and Tis lesions, respectively. However, in muscle-invasive BCa (≥T2), the sensitivity was 74.03%, indicating that NMP22 BladderChek was more sensitive to more serious diseases. Likewise, when considering tumor grade-specific sensitivity, low-grade (G1) tumors were detected with 44.16% sensitivity, G2 with 56.25% sensitivity, and high-grade (G3) with 67.34% sensitivity. These findings are consistent with its usefulness in the detection of more severe BCa but its lesser usefulness in the detection of early stage disease.

Subgroup analysis identified possible heterogeneity in diagnostic accuracy based on ethnicity and recurrence status. The test was more sensitive in Asians than in Caucasians, but the cause of this heterogeneity is not known. Genetic susceptibility, environmental exposure, and lifestyle differences are possible contributing factors, but further studies are required to confirm these findings. The test was also more sensitive for recurrent bladder cancer patients than for the first diagnosis; therefore, it was useful in disease follow-up and surveillance.

One of the primary benefits of the NMP22 BladderChek test is its fast point-of-care (POC) use, which eliminates the need for specialized laboratory equipment. Its high specificity (88%) ensures that false positives are rare, making it a viable test for ruling out BCa in individuals who test negative. False positives can still occur, particularly among individuals with hematuria, urinary tract infections, or inflammation, but at a lower incidence than that of other urine biomarkers [34].

Compared to other non-invasive BCa tests, the NMP22 BladderChek demonstrated a moderate balance between sensitivity and specificity. It is more sensitive than urine cytology, particularly for high-grade tumors, but less sensitive than UroVysion FISH, which detects chromosomal abnormalities in BCa. 

The Bladder Tumor Antigen (BTA) Stat test is another marker that is used on a regular basis and is more likely to provide false positives because of its sensitivity to inflammatory disorders. Therefore, NMP22 is a more specific option. However, no direct cost-effectiveness comparisons have been conducted to establish the financial impact of employing NMP22 in clinical practice [63].

While the NMP22 BladderChek test has the potential to be a cost-effective, fast, and user-friendly tool for detecting and monitoring high-risk BCa, its intermediate sensitivity (56%) remains a significant limitation. This limits its use as a solitary test; therefore, it needs to be used in conjunction with other diagnostic modalities such as urine cytology, cystoscopy, or FISH. Another major limitation is the heterogeneity of test performance across different patient populations, tumor types, and study designs. Furthermore, the meta-analysis did not consider NMP22’s external validity across different clinical practices, that is, primary care vs. specialty urology clinics, or patient demographic heterogeneity [34].

Subsequent studies should aim to improve the sensitivity of NMP22 by integrating it with other biomarkers, optimizing cutoff levels in different patient populations, and proving its effectiveness across ethnic groups. Further investigation into AI-based urine cytology techniques may improve the accuracy and supplement NMP22-based diagnosis. Further prospective multicenter studies should be performed to maximize its use in daily clinical practice and optimize diagnostic methods [10].

#### 3.2.9. ADXBLADDER

Minichromosome maintenance 5 (MCM5) is an indicator of DNA replication in cancer cells and proliferating cells, including BCa cells. MCM5 has been proposed as a marker for urine test detection and subsequent BCa. However, studies on its implementation are still underway. A comprehensive review and meta-analysis of eight prospective trials comprising 5114 patients revealed that MCM5 had a sensitivity of 66% and a specificity of 72%, with an AUC of 0.74, indicating reasonable diagnostic accuracy [64]. The positive likelihood ratio was 2.28, and the negative likelihood ratio was 0.50, suggesting that, while a negative test reduces the probability of bladder cancer, it does not completely rule it out. The diagnostic odds ratio (DOR) was 9.29, demonstrating a moderate ability to differentiate between cancerous and non-cancerous conditions. However, no direct statistical comparisons (*p*-values) were performed to assess the relevance of these findings in relation to other biomarkers. This study included an evaluation of ADXBLADDER, a commercially available ELISA-based test for detecting MCM5 in urine. A subgroup study of five investigations of 3000 patients utilizing ADXBLADDER revealed a sensitivity of 61% and specificity of 67%, which was somewhat lower than that of MCM5. This shows that ADXBLADDER may not be the most effective approach for detecting MCM5 compared to immunofluorometric assays and quantitative PCR-based methods, but further direct comparisons are required to validate this.

The diagnostic performance of MCM5 varies depending on the clinical context and tumor characteristics. MCM5 performed better in primary bladder cancer diagnosis, with a sensitivity of 74% and a specificity of 78%, making it a potentially helpful tool for symptomatic patients undergoing initial examination. However, its effectiveness in bladder cancer monitoring was poor, with a sensitivity of 58% and a specificity of 61%, indicating limited value for detecting recurrences.

The MCM5 demonstrated greater sensitivity (79%) and specificity (82%), indicating that it might be beneficial for screening aggressive BCa. In contrast, MCM5 sensitivity for low-grade tumors was significantly low at 50%, but its specificity remained high at 79%. These findings suggest that MCM5 is more reliable for high-grade tumors, but less effective in detecting low-grade BCa, a limitation shared by many other urinary biomarkers.

The study also compared MCM5 to urine cytology, a commonly used, yet imprecise, diagnostic approach. A pooled study of five trials found that urine cytology had a sensitivity of only 31% but a high specificity of 96%, with an AUC of 0.84. These findings demonstrate that, although urine cytology is highly specific, it lacks sensitivity, rendering it unsuitable for early cancer diagnosis.

MCM5 revealed a much greater sensitivity than urine cytology but poorer specificity. However, the lack of direct statistical comparisons (e.g., *p*-values and effect sizes) makes it impossible to assess the importance of these differences.

In addition to urine cytology, MCM5 has also been suggested as an adjunct to other urinary markers to improve the overall sensitivity. A combination panel of MCM5 and NMP22 showed 95% sensitivity and 72% specificity, suggesting a potential multi-marker strategy for BCa detection. However, further validation in large-scale, prospective research is needed before this combination can be used in clinical practice.

Furthermore, new urine biomarkers, such as Bladder EpiCheck and Xpert Bladder Cancer Monitor, have been investigated for lengthening cystoscopy intervals during BCa surveillance. Future studies should compare MCM5 with newer biomarkers to establish their diagnostic utility [65].

The therapeutic implications of our findings indicate that MCM5 is a potential but imprecise biomarker for BCa diagnosis, particularly in symptomatic patients. Although its greater sensitivity than urine cytology makes it beneficial for initial diagnosis, its intermediate performance in monitoring settings suggests that it cannot completely replace cystoscopy [66].

One of the significant benefits of ADXBLADDER is that it is non-invasive and can therefore be employed for POC diagnosis. However, its low sensitivity for low-grade cancers indicates that it is not as effective as a standalone diagnostic test. ADXBLADDER’s high negative predictive value shows that it could be beneficial in ruling out unnecessary cystoscopies, particularly in patients with high-grade malignancies; however, this must be confirmed [67].

Despite its potential, the meta-analysis had some limitations. The detection methods for MCM5 in the included studies were different, which introduced heterogeneity to the pooled analysis. Some studies used ELISA-based ADXBLADDER, while others used immunofluorometry or quantitative PCR, which may introduce variability in test accuracy. Furthermore, differences in cutoff values across studies may have contributed to differences in test performance. The fact that there were both diagnostic and surveillance settings makes pooled measures of sensitivity and specificity difficult to assess, and stratification is necessary to confirm the effectiveness of MCM5 in both settings [64].

Additionally, insufficient information is available regarding intravesical medication effects, such as BCG treatment, on MCM5 detection. This is especially significant in surveillance settings, where discrimination between true recurrences and therapy-altered changes must be made [68].

Detection of MCM5 in urine has intermediate diagnostic precision for BCa, particularly for high-grade tumors and the primary diagnosis of symptomatic disease. However, as it is less sensitive to low-grade malignancies and has inconsistent utility in surveillance settings, it is not yet a replacement for cystoscopy [64].

Although ADXBLADDER offers a highly negative predictive value, it is not sensitive enough to diagnose early BCa. MCM5 combined with other urinary markers, such as NMP22 and UroVysion, can significantly enhance its clinical usefulness and result in a less expensive and more sensitive bladder cancer diagnosis and monitoring procedure [69].

Future research should include the optimization of test performance, practice standardization, and integration of MCM5 with multi-marker panels to optimize diagnostic accuracy and prevent invasive diagnostic testing. There is also a need for a cost-effectiveness analysis to ascertain whether MCM5-based testing is cost-effective compared to existing diagnostic practices [70].

#### 3.2.10. UroSEEK

UroSEEK is a urine-based molecular diagnostic test that detects genetic alterations related to BCa. However, its therapeutic use requires further confirmation. It has three components: TERTSeqS, which detects TERT promoter mutations prevalent in bladder cancer; UroSeqS, a multiplex PCR-based assay detecting ten other genes involved in urothelial carcinoma [fibroblast growth factor receptor (*FGFR3*), *PIK3CA*, *TP53*, Harvey rat sarcoma viral oncogene homolog (*HRAS*), Kirsten rat sarcoma viral oncogene homolog (*KRAS*), Erb-B2 receptor tyrosine kinase 2 (*ERBB2*), *CDKN2A*, mesenchymal–epithelial transition factor (*MET*), mixed lineage leukemia (*MLL*), and *VHL*]; and FastSeqS, which detects aneuploidy. These components improve the detection of BCa in both early and late stages, although further comparative studies with existing diagnostic approaches are required to demonstrate their effectiveness [71].

A multicenter study examined 527 bladder urothelial carcinoma patients, with 373 non-invasive and 154 invasive tumors, from four academic institutions in four different countries [72]. Tumor samples were collected from transurethral resections or cystectomy specimens, and their mutational profiles were analyzed using UroSEEK. The total detection rate was 92%, with at least one genetic change found in the majority of cancers. TERT promoter mutations were found in 70% of the cases, with the most common modification being g.1295228C>T, followed by g.1295250C>T. Of special interest were low-grade non-invasive papillary carcinomas, in which *TERT* promoter mutations were more common (77%) than in carcinoma in situ and high-grade non-invasive cancer (65%), a statistically significant result (*p* = 0.017). However, no direct comparison has been made between UroSEEK and other urine-based molecular tests; thus, the relative clinical significance could not be established.

A mutation-screening study of 10 additional genes in UroSeqS revealed unique patterns related to tumor grade and stage. *FGFR3* and *PIK3CA* mutations were more common in low-grade noninvasive tumors than in high-grade and invasive tumors (*p* < 0.0001), whereas *TP53* mutations were more common in high-grade and muscle-invasive bladder malignancies (*p* < 0.0001). In addition, *TP53* and *CDKN2A* mutations were more common in muscle-invasive bladder cancer than in pT1 tumors (*p* = 0.005 and *p* = 0.035, respectively). While these findings are in agreement with previous studies associating *FGFR3* alterations with low-grade and *TP53* mutations with invasive cancers, a comparison with existing molecular tests such as UroVysion or Bladder EpiCheck must be made to assess the therapeutic benefit of UroSEEK [72].

The most significant aspect of the study was the analysis of sequential tumor samples from 36 patients to determine the stability of mutational profiles during disease development. *TERT* promoter mutations were consistently found in more than one tumor sample from the same patient in 22 of the 29 cases, suggesting that these mutations are an early and persistent feature of bladder cancer. However, *FGFR3* mutations exhibit variability across sequential tumors, indicating possible tumor heterogeneity in disease progression. These findings suggest that UroSEEK may be useful for longitudinal monitoring; however, its ability to predict recurrence and progression requires further investigation.

The present study evaluated the prognostic utility of UroSEEK for recurrence and progression. Among 303 patients with adequate follow-up data, 94% of tumors that later recurred had at least one mutation in the UroSEEK panel, which was similar to the 95% of tumors that did not recur (*p* = 0.784). Although UroSEEK positivity did not substantially predict recurrence, tumors that advanced to a higher stage were less likely to have UroSEEK mutations than non-progressing tumors (81% vs. 96%, *p* = 0.016). This shows that UroSEEK has excellent diagnostic sensitivity but a low predictive value for tumor development, indicating the need for additional biomarkers or clinical factors to improve prognostic accuracy.

The detection rate of UroSEEK was compared to that of urine cytology. Previous research has shown that 100% of low-grade noninvasive papillary carcinomas are undiagnosable by urine cytology, but UroSEEK can detect mutations in 67% of these cases. However, although UroSEEK is more sensitive for low-grade cancer, its specificity compared with urine cytology remains to be assessed, and judgments must be withheld regarding its full diagnostic capability.

The relative sensitivity and specificity of UroSEEK are uncertain when compared to other urine biomarkers such as UroVysion and Bladder EpiCheck. Future research should directly compare UroSEEK with known tests in terms of diagnostic accuracy, cost-effectiveness, and clinical viability [73].

Despite these promising findings, this study has several limitations. The mutation prevalence varied compared to prior sequencing studies, possibly due to differences in targeted regions and sequencing depth. The group had historical tumor specimens collected between 1991 and 2016, which can harbor storage of sample-related biases, as well as biases due to the development of therapeutic care processes. There is limited information regarding UroSEEK performance across various populations, including ethnicity, smoking status, and age-related variables that may affect the prevalence of mutations. There was no information on false negatives and false positives; therefore, it was difficult to estimate how well UroSEEK works in everyday screening practice. Whether cost-effectiveness and accessibility are appropriate for UroSEEK compared to routine urine cytology or other molecular tests is questionable. Future studies should evaluate the therapeutic benefit of combining UroSEEK with other urine markers, establish whether UroSEEK can eliminate unnecessary cystoscopies, and confirm these findings in large prospective studies [72]. UroSEEK was diagnostically successful and detected genetic alterations in over 90% of bladder cancers across the majority of the histological subtypes. The high mutation frequency of the TERT promoter in low-grade noninvasive cancer supports its application for early detection and the addition of other oncogenic mutations enhances its sensitivity for high-grade and muscle-invasive BCa.

However, its prognostic utility in predicting progression is unknown, and its specificity in comparison with urine cytology and other molecular tests has not been clearly demonstrated. Future research should compare the performance of UroSEEK with known urine biomarkers, assess its cost-effectiveness, and establish whether it can minimize the need for invasive cystoscopy in bladder cancer surveillance.

#### 3.2.11. AssureMDx

AssureMDx is a urine-based molecular test developed for the early detection of BCa and monitoring of tumor recurrence, particularly in high-risk patients. It is a noninvasive diagnostic tool that analyzes DNA methylation markers, mutational changes, and chromosomal abnormalities to improve the detection of UCa. While AssureMDx increases the detection levels of high-grade BCa, it is not as sensitive to low-grade tumors; thus, its use in conjunction with other diagnostic agents is required [74]. The test is especially useful in patients with hematuria, as well as in those who are being actively monitored for BCa recurrence. Compared with other conventional techniques, such as urine cytology, which has excellent specificity (>90%) but low sensitivity (30–60%), and invasive cystoscopy, AssureMDx offers a molecular alternative that can potentially augment the detection rate, particularly in high-risk individuals. However, it is not meant to replace cystoscopy because histological evidence is still required for final diagnosis and staging.

AssureMDx detects gene mutations and DNA methylation patterns that are abnormal in bladder cancer. The test checks for mutations in *TERT*, *FGFR3*, and *HRAS* and changes in three important epigenetic markers [*OTX1*, One Cut Homeobox 2 (*ONECUT2*), and *TWIST1*]. Molecular changes are characteristic of urothelial carcinoma but have varying specificity depending on the type of patient and the stage of the tumor. By analyzing exfoliated urinary cells, AssureMDx enhances diagnostic accuracy, but false positives may occur due to non-malignant conditions, such as inflammation or benign urothelial hyperplasia [75].

The test operates on a methylation-specific PCR (MSP) platform, which allows for the quantification of DNA methylation levels in genes associated with tumor progression and recurrence. However, its dependence on MSP may influence the sensitivity of tumor detection with heterogeneous methylation patterns, which should be taken into account during interpretation.

Clinical trials have demonstrated that AssureMDx is more sensitive and has a higher negative predictive value (NPV) than noninvasive screening and urine cytology. In a large prospective study, AssureMDx was 93% sensitive for detecting high-grade urothelial carcinoma (HGUC) and had an NPV of 99%, indicating that a negative test reliably excludes bladder cancer. However, the test sensitivity for low-grade tumors is significantly lower (~40–60%) and thus it is less helpful for detecting early stage, low-grade disease [76].

Urine cytology is less sensitive (30–60%) for detecting bladder cancer but is not used for the detection of low-grade bladder cancer. AssureMDx’s high NPV suggests that it may be helpful in ruling out malignancy in some patients, lowering the number of unnecessary cystoscopies [74]. However, it should be evaluated in conjunction with clinical risk factors, as no single molecular test can completely replace the standard-of-care diagnostic techniques. AssureMDx’s ability to stratify patients based on risk allows for a more tailored approach to BCa diagnosis and care. A patient with a positive AssureMDx result has a high likelihood of BCa and should undergo further assessment using cystoscopy and, where appropriate, biopsy. A negative result, on the other hand, is definite proof that no cancer exists and allows practitioners to avoid invasive examinations in appropriate patients. AssureMDx has been demonstrated to be useful in following BCa in patients with a history of recurrent NMIBC. Others have discovered that AssureMDx is capable of identifying molecular alterations that point to recurrence even before tumor detection on cystoscopy. However, additional data are needed to evaluate its capacity to predict recurrence with high precision, as false positives from nonmalignant diseases continue to be an issue.

Although AssureMDx is more sensitive than urine cytology, research is still being conducted on how well it performs compared to other urinary biomarkers, including UroVysion (a FISH-based assay), Bladder EpiCheck (a DNA methylation panel), and CxBladder (an mRNA-based test). Although commonly used for monitoring, UroVysion FISH is not as sensitive for low-grade tumors but can detect chromosomal changes related to bladder cancer. Bladder EpiCheck, another DNA methylation biomarker, was reported to be highly specific, but a direct comparison with AssureMDx is not available [77].

Follow-up comparison studies will have to establish the optimal functioning of AssureMDx in the general context of molecular BCa diagnoses. While it is useful, AssureMDx is not without limitations. It is less sensitive than urine cytology and may yield false-positive findings in patients with benign urinary masses or inflammatory disorders. This may lessen the need for unnecessary cystoscopies, but does not ensure that they will not be performed, as histological and visual confirmation are still required. The cost-effectiveness of this test compared with other molecular tests and standard-of-care diagnostic tests has not been widely studied [78].

More real-world studies are needed to evaluate its therapeutic impact in a wide range of patient populations, including those of age, ethnicity, and concomitant disorders. No data have been reported on the influence of intravesical treatment (e.g., BCG treatment) on test performance, which may be useful for surveillance [75].

AssureMDx is a significant advance in BCa diagnosis beyond traditional invasive approaches, with superior sensitivity, superior negative predictive value, and the potential to decrease reliance on invasive treatment in some instances. However, limitations in specificity, reduced sensitivity for low-stage tumors, and the need for further comparison trials underscore the need to incorporate it into a multimodal diagnostic regimen rather than as an isolated diagnosis [78].

Future studies should focus on improving its specificity, enhancing its role in monitoring, and comparing its cost-effectiveness with that of other urine biomarkers. Combining AssureMDx with AI-assisted diagnostic tools or multi-marker panels might also improve its clinical relevance, making BCa diagnosis and monitoring more efficient and patient-friendly [79].

## 4. Molecular Tests in the Laboratory Diagnosis of Bladder Cancer

**Figure 3:**
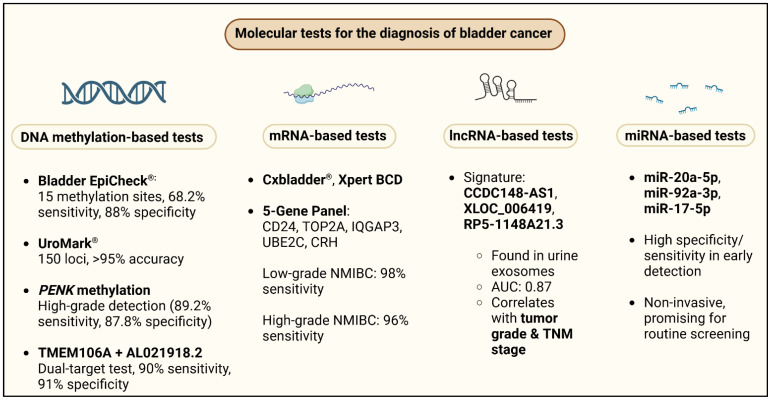
Overview of urine-based molecular biomarkers for bladder cancer diagnosis.

Over the past decade, many molecular assays have been evaluated and have resulted in considerable advances in the diagnosis of urothelial carcinoma compared to urinary cytopathology (Figure 3). However, these methods are not simpler, more rapid, or less costly than the currently available methods [80].

### 4.1. DNA Methylation

Epigenetic changes, in the form of DNA methylation, play a key role in BCa development because they regulate gene expression without changing the underlying DNA sequence. Several panels of DNA methylation based on urine have been established with high diagnostic accuracy. Specifically, Bladder EpiCheck^®^ analyzes 15 methylated genes and has been demonstrated to have 68.2% sensitivity and 88% specificity for the detection of BC, whereas UroMark, which analyzes 150 methylation sites by next-generation sequencing, has up to 98% sensitivity and 97% specificity. BladMetrix and Bladder CARE, among others, have reached more than 90% sensitivity, validating that the utilization of methylation biomarkers is worth considering in diagnostic applications. In addition to DNA methylation, ncRNAs (miRNAs, lncRNAs, and circRNAs) have shown substantial promise as urine biomarkers. Several miRNAs, including miR-20a-5p, miR-92a-3p, and miR-17-5p, have shown greater than 90% sensitivity and specificity for identifying BC, whereas lncRNAs such as *UCA1-201*, HOX transcript antisense RNA (*HOTAIR*), and metastasis associated lung adenocarcinoma transcript 1 (*MALAT1*) have comparable diagnostic accuracy. Circular RNAs, albeit understudied, are emerging as useful indicators, with circRNA-0071196 showing 87.5% sensitivity and 85% specificity for BCa detection. 

Urination is noninvasive for the collection of urine, making such RNA-based biomarkers perfect for routine screening and follow-up. In addition to early detection, epigenetic markers are important for prognosis. DNA methylation patterns such as protein tyrosine kinase 2 (*PTK2*) hypermethylation have been found to be associated with overall survival, whereas bladder cancer associated protein (*BLCAP*) hypomethylation is strongly associated with recurrence and cancer-specific death risk. Similarly, ncRNAs such as miR-21 and lncRNA *XIST* have been implicated in tumor growth and drug resistance, offering important insights into personalized treatment protocols. The incorporation of epigenetic markers into the clinic has the potential to transform BCa care by facilitating early diagnosis, risk stratification, and monitoring of individualized therapy. Urine analyses of DNA methylation and ncRNA expression are a noninvasive and highly sensitive substitutes for standard cystoscopy and urine cytology. As more large-scale clinical trials verify these biomarkers, their implementation in standard screening protocols has the potential to further improve diagnostic precision, decrease the need for invasive tests, and improve patient outcomes. Further technological innovations in liquid biopsy and standardization of the test will be required to better leverage epigenetic biomarkers in the management of BCa in the future [81].

Given their association with tumorigenesis and progression, DNA methylation biomarkers have emerged as promising tools for early cancer detection. DNA methylation with hypermethylated and hypomethylated regions is implicated in bladder cancer [82,83,84]. The methylation of proenkephalin (*PENK*) in urinary sediments is a new biomarker for early BCa diagnosis [85,86]. Other studies have established that a urine test for *PENK* methylation is sensitive and specific for identifying BCa in patients with hematuria [87,88]. Nevertheless, these findings are constrained by the single-center study designs and the small sample sizes. In a large prospective multicenter study conducted at ten South Korean medical sites, the sensitivity and specificity of the *PENK* methylation test for bladder cancer detection, particularly high-grade or invasive types, were investigated, and its diagnostic accuracy was compared to that of NMP22 and urine cytology tests. Of the 1099 enrollees, 219 had BCa, including 176 patients with invasive or high-grade disease. The methylation test on urinary *PENK* was 89.2% sensitive (95% CI, 84.6–93.8%) and 87.8% specific (95% CI, 85.6–89.9%) for the detection of invasive or high-grade BCa. For all BCas, the test had a sensitivity of 78.1% (95% CI, 72.6–83.6%) and a specificity of 88.8% (95% CI, 86.7–90.8%). The PPV for high-grade or invasive bladder cancer was 61.3%, while the NPV was 97.6%, indicating that the test is highly effective in ruling out disease, but less reliable for confirming positive cases. In comparison, the NMP22 test exhibited lower sensitivity, detecting only 51.5% of high-grade or invasive BCa cases, while the urine cytology test was even less sensitive, identifying only 39.7%. However, urine cytology showed the highest specificity (99.5%), indicating that it had the lowest false positive rate. When combining the *PENK* methylation test with the NMP22 or urine cytology test, the sensitivity slightly increased, but the specificity declined, suggesting that the *PENK* test alone provides the best balance of sensitivity and specificity. 

This study highlights that DNA methylation changes play a crucial role in BCa development, with methylation markers such as *PENK* offering superior diagnostic performance compared to protein-based urinary biomarkers. Unlike low-grade BCa, which has slow progression, high-grade or invasive BCa requires early detection for better clinical management, making a high-sensitivity test essential. Although the *PENK* methylation test significantly outperformed both NMP22 and urine cytology in terms of sensitivity, its positive predictive value remains suboptimal, which means that a positive test result should still be confirmed by cystoscopy [89].

Using whole-genome bisulfite sequencing (WGBS) of BCa tissues and paired normal tissues, differentially methylated regions associated with UC were identified. By integrating WGBS data with The Cancer Genome Atlas (TCGA) urothelial bladder cancer dataset, Transmembrane Protein 106A (TMEM106A) was identified as a novel UC biomarker. To enhance the diagnostic accuracy, TMEM106A was combined with a previously established UC methylation biomarker, AL021918.2, to form a dual-target urine test. This panel was evaluated using voided urine samples from 224 UCa patients and 419 controls, which included individuals with non-malignant urinary diseases and healthy donors. This study revealed significant hypomethylation in UC tissues compared with normal tissues. Differential methylation analysis identified multiple aberrant CpG sites, with TMEM106A emerging as a key biomarker owing to its high specificity and robust performance in distinguishing cancerous from non-cancerous tissues. When tested on urine samples, the dual-target assay demonstrated remarkable diagnostic efficacy. In the training set, it achieved a sensitivity of 89.0%, a specificity of 92.9%, and an AUC of 0.941. The validation set yielded similar results, with a sensitivity, specificity, and AUC of 90.0%, 91.1%, and 0.922, respectively. The test performed particularly well in detecting early-stage and low-grade UC, which is often missed by traditional diagnostic methods. The dual-target test not only showed high sensitivity and specificity, but also outperformed existing urine-based biomarkers, such as urine cytology and other DNA methylation assays. Unlike multi-gene panels, which may compromise specificity, the selection of just two highly specific biomarkers optimized the balance between sensitivity and accuracy. The qMSP method used for detection was cost-effective, scalable, and suitable for clinical implementation. 

Despite its promising results, this study has several limitations. The cohort was primarily composed of patients with UBC, with a smaller number of UTUC cases, necessitating further validation in a larger and more diverse population. Additionally, while the test showed high specificity, its false-positive rate, though lower than that of other methylation-based panels, still warrants further refinement. Future studies should explore their performance in terms of longitudinal monitoring and recurrence detection [90].

### 4.2. mRNA

A recent study [91] assessed the diagnostic value of a tumor progression-related mRNA panel in urine samples from patients with NMIBC, hematuria caused by nonmalignant diseases, and healthy controls. The study included 129 participants: 67 patients with NMIBC, 31 with hematuria caused by benign urological disorders, and 31 healthy controls. To identify highly dysregulated mRNAs, urine samples from all participants were subjected to quantitative RT–qPCR. Ten tumor-related mRNAs were chosen for analysis based on their roles in tumor growth, proliferation, and progression. The results showed that carbonic anhydrase 9 (*CA9*), *CDK1*, cluster of differentiation 24 (*CD24*), *TERT*, centrosomal protein 55 (*CEP55*), *TOP2A*, IQ motif containing GTPase activating protein 3 (*IQGAP3*), *UBE2C*, and corticotropin releasing hormone (*CRH*) were highly upregulated in patients with NMIBC compared to a healthy population. *CD24*, *TOP2A*, *IQGAP3*, *UBE2C*, and *CRH* mRNA levels were also significantly higher in patients with NMIBC than in those with hematuria, which may indicate that they could be used as discriminative biomarkers for BCa detection compared to urological diseases. 

To evaluate the diagnostic accuracy of these markers, a five-gene panel (*CD24*, *TOP2A*, *IQGAP3*, *UBE2C*, and *CRH*) was used. Compared to the healthy controls and the hematuria group in discriminating low-grade NMIBC, the panel was 98% sensitive (95% CI: X-X%) and had 100% and 90% specificity, respectively. The panel showed a sensitivity of 96% (95% CI: X-X%) and 100% for high-grade NMIBC, as well as a specificity of 100% and 83% when compared to healthy controls and the hematuria group, respectively. While these results suggest excellent diagnostic potential, further validation in larger independent cohorts is required to confirm reproducibility and reduce the risk of overfitting.

Although this mRNA-based panel is promising, it should be compared with established urinary biomarkers for the detection of NMIBC. The CxBladder, another mRNA-based test, is a multi-gene marker assay that is extensively used in clinical settings. UroVysion, a FISH-based test, detects chromosomal alterations that are associated with BCa and is typically applied to surveillance but is not as sensitive for low-grade tumors. Bladder EpiCheck, which analyzes DNA methylation patterns, offers high specificity but variable sensitivity across different tumor grades. Direct statistical comparisons between this five-gene panel and existing biomarkers are necessary to determine their clinical advantages and whether they provide complementary or superior diagnostic value [92].

While encouraging, there are several caveats to consider. The trial was only performed on 129 patients; therefore, it lacked statistical power and external validity. High sensitivity and specificity rates are potential markers for overfitting; hence, validation in independent, multicenter cohorts is required. Furthermore, while the panel had very good specificity, the false-positive rate in patients with inflammatory conditions or benign urological disease is unknown. False negatives can also be caused by tumor heterogeneity in mRNA expression, which can affect the detection of early lesions. The clinical use of this panel also needs to be cost-effective compared to existing urine-based assays and standardized in the laboratory to provide reproducible performance of the tests on various platforms [91].

In general, this mRNA-containing urine biomarker panel had excellent diagnostic performance in detecting NMIBC with high sensitivity and specificity for low-grade and high-grade tumors. However, further validation in a larger, independent series is required to determine its robustness and clinical utility. Future studies should contrast this panel with existing urinary biomarkers, ascertain its cost-effectiveness, and study its real-world performance in diverse groups of patients. Merging this panel with diagnostic devices aided by artificial intelligence or multi-marker panels will further optimize its clinical application in NMIBC detection and monitoring [93].

### 4.3. LncRNA

The urine exosomal long non-coding RNA (lncRNA) signature is a promising noninvasive biomarker for the early detection of NMIBC. To investigate its diagnostic potential, microarray differential expression profiling between NMIBC patients and normal donor urine exosomes was performed using quantitative real-time polymerase chain reaction (qRT–PCR) validation [94]. Exosomes are small extracellular vesicles that cells, including tumor cells, secrete into body fluids; they contain RNA, proteins, and lipids and are therefore stable carriers of molecular biomarkers and valuable tools for liquid biopsy.

This study followed a structured validation process involving three independent phases: bladder cancer (BCa) cell lines, culture fluids, and a cohort of 200 patients with NMIBC. This strategy revealed a three-lncRNA signature, CCDC148-antisense RNA 1(AS1), XLOC_006419, and reference sequence 5 (RP5)-1148A21.3, which was highly overexpressed in NMIBC patients compared to normal controls. ROC curve analysis also attested to its very high diagnostic accuracy, with AUCs of 0.873, 0.825, and 0.834 in the training, validation, and double-blind validation stages, respectively. These data indicate a strong discriminative capacity for the differentiation of NMIBC from normal controls, which is significantly better than that of conventional urinary cytology (*p* < 0.0001). However, despite these encouraging results, the findings must be confirmed in independent multicenter cohorts before its clinical utility and reproducibility can be ascertained.

In addition to their potential as diagnostic markers, lncRNAs have also been shown to be associated with disease severity. Overexpression of CCDC148-AS1 was strongly associated with a higher tumor grade (*p* < 0.001), suggesting its role in tumor development. CCDC148-AS1 and XLOC_006419 were also strongly associated with tumor–node–metastasis (TNM) stage (*p* = 0.004 and *p* = 0.031, respectively); therefore, they may also be prognostic markers for bladder cancer development. Further experiments demonstrated that these lncRNAs are actively secreted by BCa cells and packaged within exosomes, a feature that enhances their stability in urine and reinforces their potential as reliable liquid biopsy biomarkers.

Although this lncRNA signature demonstrated strong diagnostic potential, its performance must be assessed in comparison with existing urinary biomarkers used in NMIBC detection. Clinically validated tests such as CxBladder (a broad clinical practice mRNA-based assay), UroVysion (a fluorescence in situ hybridization chromosomal alteration test), and Bladder EpiCheck (a urine DNA methylation test) have already been clinically validated for use. Head-to-head comparisons of this three-lncRNA panel with validated biomarkers must be conducted to determine its relative clinical utility and whether it is better or complementary to their current diagnostic utility [95].

Despite its encouraging outcomes, there are various limitations that must be considered before this panel can be applied in standard clinical practice. A total of 200 NMIBC patients were studied, which is considerable but still requires further replication in larger multicenter trials to establish reproducibility. High AUC values are suggestive of good performance; however, clinical practice can manifest variability, especially in heterogeneous patient groups. Furthermore, the potential for false positives, where lncRNA levels may be influenced by non-malignant conditions such as inflammation or benign urologic pathology, remains. False negatives could also occur in low exosomal RNA-secreting tumors, which could diminish the panel’s sensitivity for early NMIBC detection [94].

A second primary consideration in clinical translation is the convenience of isolating and quantifying exosomal RNA. Whether screening for exosomal lncRNAs is cost-effective relative to other urine-based biomarkers remains unclear. Most critically, standardization of laboratory practices must be ensured to ensure reproducibility and consistent test performance. With these hurdles eliminated, exosomal lncRNA signatures can revolutionize NMIBC diagnosis with very sensitive, noninvasive testing, eliminating the need for existing screening modalities.

In conclusion, this study created a three-lncRNA exosomal signature with high diagnostic accuracy, sensitivity and specificity for the detection of NMIBC. The included lncRNAs also correlate with tumor grade and stage, suggesting that they can serve as biomarkers for early disease detection and monitoring of disease progression. However, to translate this biomarker panel into clinical practice, further validation in larger cohorts, head-to-head comparisons with established urinary biomarkers, and cost–utility and practicality analyses are required. Future research should focus on increasing test standardization, exploring multi-biomarker integration, and establishing its clinical utility in real-world settings. Combining exosomal biomarkers with AI-driven analyses can further increase their diagnostic power, ultimately improving NMIBC detection and patient care.

### 4.4. miRNA

MicroRNAs (miRNAs) are emerging as new noninvasive BCa biomarkers for the sensitive and specific detection of disease as well as for therapeutic monitoring. Together with established detection strategies, they may advance detection rates earlier and minimize invasive cystoscopy interventions. While these results are encouraging, miRNA testing remains unstandardized for the clinic, and multicenter validation experiments need to be conducted before the test can be used widely [96].

A systematic review of 21 studies [97] evaluated the diagnostic discrimination of different miRNAs and concluded that there was a high discriminatory accuracy in the majority of candidates. ROC curve analysis demonstrated that individual miRNAs such as miR-9, miR-34a, and miR-203 achieved an AUC of 0.92, with a sensitivity of 93.3% and a specificity of 80%. Another combination of miR-96-5p and miR-183-5p yielded an AUC of 0.88, with a sensitivity of 88.2% and specificity of 87.8%. Multi-miRNA panels were also superior, with some of them having an AUC of 0.96, sensitivity of 87.8%, and specificity of 93.3%, which is better than most of the existing diagnostic tests. In addition to their diagnostic value, miRNAs have also proven to be of prognostic value, with some of them, such as miR-155-5p, showing correlations with tumor stage, lymph node metastasis, and survival. Furthermore, differential miRNA expression following surgical intervention indicates their potential use in monitoring therapeutic responses and the risk of recurrence.

The integration of miRNAs with traditional diagnostic methods has been shown to provide additional sensitivity for detection. Diagnostic efficacy was greatly improved when miR-192 was integrated with ultrasound, with 93.2% sensitivity and 76.7% specificity compared to cystoscopy. These findings illustrate the promise of using a combination of miRNAs and imaging and cytology for enhanced bladder cancer detection, which is still noninvasive. However, before these multimodal strategies can be applied clinically, standardization of miRNA detection techniques and further validation in independent groups need to be accomplished.

In addition to miRNAs, a multi-marker approach with protein-based and DNA methylation biomarkers is another promising avenue for noninvasive BCa detection. A 1119 urine sample study combined C-X-C Motif Chemokine Ligand 16 (CXCL16) and transforming growth factor beta induced protein (TGFBI) as protein biomarkers, with CpG sites arachidonate 5-lipoxygenase (ALOX5), TRPS1, and an intergenic region on chromosome 16 as DNA methylation markers. CXCL16 alone showed a sensitivity of 31% and specificity of 94%, whereas TGFBI showed a sensitivity of 56% and specificity of 85%. The inclusion of DNA methylation markers improved the detection of urothelial carcinoma in men with a sensitivity of 54% and specificity of 94%. Notably, when all five markers were tested in combination, bladder cancer detection in a heterogeneous population, encompassing cancer-free individuals and individuals with other urological or gynecological cancers, achieved 97% specificity and 72% sensitivity. These findings demonstrate the effectiveness of multi-marker approaches in maximizing diagnostic accuracy with few false positives and unnecessary cystoscopies [98].

An additional study [99] examined adenomatous polyposis coli (*APC*) promoter methylation as a potential urinary biomarker of bladder cancer. The analysis using urine-derived DNA from 50 BCa patients and DNA from 50 controls and revealed a significant difference in *APC* promoter methylation between the patient and control groups (*p* < 0.001). Specifically, 34 of 50 (68%) BCa cases were associated with *APC* methylation, compared to 8 of 50 (16%) controls. There were increased rates of *APC* methylation in high-grade tumors (*p* = 0.048) and, therefore, the implication of a correlation with the tumor’s aggressiveness, although no statistical correlation was observed between tumor stage and *APC* methylation rates. In addition, a strong correlation was observed between smoking status and *APC* methylation (*p* < 0.001), confirming previous evidence of a relationship between tobacco exposure and epigenetic alterations in BCa. These findings indicate that *APC* promoter methylation could be an early detection biomarker, rather than a marker of disease progression. Future studies should investigate whether the combination of APC methylation with other molecular markers could offer enhanced diagnostic sensitivity.

Additional research has explored DNA methylation biomarkers as noninvasive urinary markers for bladder cancer detection. In a validation study of neurintin 1 (*NRN1*), galanin receptor 1 (*GALR1*), and heart and neural crest derivatives expressed 2 (*HAND2*) methylation levels in urine samples from 77 primary BCa cases and 69 controls, which were replicated in an independent series of 63 BCa patients and 71 controls, it showed good diagnostic potential. Notably, the study employed a home-based urine collection method, allowing subjects to mail their samples for testing, thus enhancing the feasibility of mass screening. The methylation levels of *NRN1*, *GALR1*, and *HAND2* were significantly higher in patients with BCa (*p* < 0.001), and when used together, these markers had an AUC of 0.94, with 84% sensitivity and 96% specificity in the training cohort. The validation group had an AUC of 0.89, sensitivity of 76%, and specificity of 93%. Decision curve analysis identified that the incorporation of these biomarkers into clinical pathways would reduce the number of cystoscopies by 20–35% depending on the clinical circumstances, which suggests that such markers could prove useful as triage markers to identify high-risk individuals and prevent invasive tests in a substantial proportion of cases [89].

In general, miRNAs, DNA methylation markers, and protein biomarkers are promising noninvasive markers for bladder cancer surveillance and detection. Multi-marker approaches, particularly the combination of miRNA, methylation, and protein biomarkers, have been shown to have high diagnostic specificity, with some panels generating AUC scores above 0.94. In addition, combining these biomarkers with imaging or urine cytology could enhance earlier detection, with the added advantage of reduced reliance on cystoscopy. However, several barriers must be cleared for clinical translation to become feasible, including possessing standardized detection technology, validation across different patient groups, and being cost-effective in large-scale screening applications. Future research would entail optimizing biomarker panels, adding artificial intelligence-based analysis, and evaluating real-world performance through multicenter clinical trials. If properly validated, such noninvasive techniques have the potential to revolutionize bladder cancer diagnosis to facilitate earlier diagnosis, reduce unnecessary procedures, and ultimately improve patient outcomes [100].

#### Uromonitor

Uromonitor is a urine-based biomarker test for bladder cancer recurrence detection that screens hotspot mutations in the *TERT*, *FGFR3*, and *KRAS* genes. This shows a promising application perspective for the surveillance of NMIBC with the potential to reduce the application of invasive cystoscopy without sacrificing high diagnostic accuracy. Despite the higher sensitivity and specificity of Uromonitor, additional multi-center validation, cost-effectiveness analysis, and stage-specific performance evaluation are required prior to its application in routine clinical practice. In a 439-patient trial receiving 528 NMIBC surveillance visits, Uromonitor was compared with standard-of-care (SOC) methods, and each patient provided a urine sample for Uromonitor analysis before cystoscopy [101]. The trial used the sensitivity, specificity, PPV, and NPV to quantify the diagnostic performance of Uromonitor for recurrence identification. It was 87% sensitive (95% CI: 74–95%), identifying 41 out of 47 recurrences and missing 6. It was 99% specific (95% CI: 98–100%) with minimal false positives. The 93% PPV (95% CI: 82–98%) confirmed that most of the positive Uromonitor tests were for recurrences, and the 99% NPV (95% CI: 97–99%) confirmed a good predictive value of excluding recurrence with a negative test. Importantly, Uromonitor also out-performed cystoscopy for specificity, with three false positives compared to 22 (32%) false positives for cystoscopy. Recurrence in the study cohort was 8.9% (n = 47), which also provides a means to validate Uromonitor’s accuracy across the NMIBC grade and stage range. The findings suggest that Uromonitor can be a valuable addition to cystoscopy, improving detection and reducing unnecessary follow-up investigations. Nevertheless, further research is needed to determine how its performance varies by NMIBC subtype, and whether it is maintained over longer surveillance periods.

Beyond Uromonitor, another genomic urine assay was evaluated in a prospective study involving 204 high-risk NMIBC (HR-NMIBC) patients under active surveillance [75]. This assay analyzed DNA mutations in *FGFR3* and *TERT*, as well as the methylation status of OTX1, using 736 paired urine samples (collected in the evening and morning) before each cystoscopy visit. Among these patients, 63 recurrences were detected during surveillance, and concomitant urine assay results were available. In cross-sectional analysis, the urine assay achieved a sensitivity of 75% (95% CI: 62.1–84.7%) and a specificity of 70% (95% CI: 66.4–73.5%), indicating that while it effectively identified recurrences, it had a higher false-positive rate compared to Uromonitor. Further statistical modeling demonstrated that *OTX1* (*p* = 0.005) and *TERT* (*p* = 0.004) were independent predictors of disease recurrence, confirming the biological significance of these markers.

Most notably, in this study, the urine assay was able to predict future recurrence. Longitudinal examination revealed that urine assay positivity robustly predicted recurrence (HR 3.5, *p* < 0.001), which suggests that individuals with positive test results must be stringently monitored for recurrence. In addition, those developing a recurrence during the study were at a greater risk for later recurrences (HR 2.1, *p* < 0.001), establishing the chronic nature of HR-NMIBC. These findings suggest that a urine test can be useful for long-term surveillance and risk stratification, with the possibility of individualized follow-up regimens.

Although both Uromonitor and the genomic urine assay demonstrated strong diagnostic performance, they differed in their strengths and clinical applications. Uromonitor was more specific (99%) than the genomic urine assay (70%) and was therefore a safer tool for preventing false positives and futile interventions. However, the genomic urine assay’s predictive ability over time suggests the potential for long-term risk stratification and tailored disease surveillance.

Despite these promising results, several issues must be resolved before these urine-based tests can be implemented for the surveillance of NMIBC in routine practice. First, additional multicenter studies are required to validate their performance in diverse patient populations and NMIBC types. Second, cost-effectiveness analyses must be conducted to balance the economic effects against conventional cystoscopy-based surveillance. Third, additional studies are warranted to optimize these tests for different NMIBC grades and stages, particularly to distinguish indolent from aggressive recurrence [102].

Given their superior diagnostic performance and ability to reduce cystoscopy numbers, these urine assays represent valuable advancements in the management of NMIBC. Provided that they can be successfully implemented in clinical practice, they can contribute to enhancing patient comfort, saving healthcare costs, and streamlining long-term disease surveillance strategies. Additional studies in the guise of randomized controlled trials, however, are needed to confirm their impact on clinical management and patient outcomes.

## 5. Spectroscopy

Surface-Enhanced Raman Scattering (SERS) is a sensitive, label-free, and molecularly specific noninvasive detection technique, making it a desirable method for BCa diagnosis. Characterization by SERS, recently via techniques such as SERSomes, makes it possible to completely profile urine metabolites in low-grade BCa patients and healthy controls and provides a deeper insight into the metabolic changes that occur with respect to BCa. With the integration of machine learning models and SERSome spectra, a study reported 89.47% diagnostic accuracy to differentiate low-grade BCa and 90% stratification accuracy to differentiate between disease grades. The method is rapid, noninvasive, cost-effective, and can be used as an ancillary tool in large-scale population screening applications. However, more stringent evaluations in larger and multicenter populations must be conducted before clinical use [103].

In another study, SERS was combined with multivariate statistical analysis to distinguish bladder cancer stages with excellent accuracy based on urine samples from healthy volunteers, patients with NMIBC, and patients with MIBC. Spectral differences revealed biochemical distinctions related to primary biomolecular constituents such as DNA/RNA, hypoxanthine, albumin, D-(+)-galactosamine, fatty acids, and amino acids, which have been implicated in BCa metabolism and growth. These spectral variations formed the basis of a highly accurate diagnostic model for the detection and staging of BC using partial least squares-discriminant analysis (PLS-LDA) as a two-step binary classifier. The model had 97.7% diagnostic accuracy for differentiating healthy individuals from BC patients and 96.3% for differentiating NMIBC from MIBC, indicating that SERS is a highly precise, noninvasive diagnostic agent [104]. A crucial aspect of this study was the identification of key Raman spectral peaks at 803, 893, 1139, 1375, and 1466 cm^−1^, which were essential for distinguishing among healthy controls, NMIBC, and MIBC patients. The researchers also maximized the spectral measurement efficiency, demonstrating that a span of 400–1600 cm^−1^ was sufficient for accurate BCa classification, with no need for unnecessary spectral sophistication. This finding is particularly advantageous for large-scale clinical applications where efficient and fast spectral gathering is critical.

Although these studies point to SERS as a possible tool for BCa staging and diagnosis, several hurdles must be overcome before widespread clinical application. First, additional multicenter validation studies are needed to confirm the reproducibility in large patient populations and NMIBC subtypes. Second, the studies did not evaluate false positives and false negatives, which are critical for understanding the potential misclassification risks, particularly in patients with benign urinary conditions. Furthermore, the performance of SERS has not been compared directly to conventional urine-based testing, such as urine cytology, UroVysion (FISH), NMP22, or CxBladder; therefore, whether SERS represents a superior diagnostic advantage is unknown [105].

Additionally, the cost-effectiveness and feasibility of the large-scale implementation of SERS must be considered. Although SERS offers high sensitivity and specificity, its practical applicability depends on the availability of standardized spectral analysis protocols, reproducibility across different laboratories, and the relative cost-effectiveness of SERS instrumentation compared to existing diagnostic protocols. Standardization of spectral interpretation data, integration of SERS into routine clinical practice workflows, and deciding on the use of AI-based spectral analysis to streamline classification models in the future are crucial [106].

In general, SERS-based urinary diagnosis has the potential to improve non-surgical staging and diagnosis of BCa because the findings have sufficient sensitivity and specificity for differentiating between NMIBC and MIBC. SERS, with its efficient machine learning algorithm and spectral efficiency, has the potential to forgo repeated invasive cystoscopy procedures, save patients, and lower healthcare expenses. However, before this approach can be put into clinical practice at the clinic, further validation studies, cost-effectiveness analysis, and comparisons with current standard-of-care techniques need to be performed. Successful validation of SERS would revolutionize the diagnosis of bladder cancer by offering a rapid, sensitive, and low-cost screening approach for disease detection and monitoring [107].

## 6. Cell Free DNA

Cell-free tumor DNA (ctDNA) has emerged as a promising biomarker for BCa diagnosis, surveillance, and personalized treatment decisions, offering a noninvasive alternative to traditional methods, such as cystoscopy and urine cytology. Recent studies have indicated that urine tumor DNA (utDNA) has 91% sensitivity and 96% specificity for the diagnosis of BCa, which is superior to conventional techniques. Elevated levels of ctDNA and utDNA are correlated with cancer progression, particularly from NMIBC to MIBC. Additionally, in radical cystectomy patients, the presence of ctDNA is associated with worse overall survival and an increased risk of recurrence, further establishing its prognostic significance. In addition, after neoadjuvant chemotherapy, ctDNA positivity confirms the identification of underlying persistence of the tumor on surgical pathology, again establishing its utility as a measure of response. However, despite these data confirming the clinical utility of ctDNA, validation in large-scale multicenter trials is necessary before its routine use [108]. Apart from its prognostic and diagnostic uses, ctDNA allows for the identification of genetic mutations that can be acted upon, enabling tailored therapy choices. For example, *FGFR3* mutations detected in ctDNA can guide the use of *FGF*R inhibitors as a targeted treatment for BCa patients. Quantifying ctDNA levels during treatment also enables the early identification of resistance mutations, with the potential to make timely therapeutic changes. Despite these advantages, drawbacks such as degradation of ctDNA, heterogeneity of tumor shedding, and standardization of detection assays remain barriers for large-scale clinical application [109].

An alternative to ctDNA analysis is circulating free DNA (cfDNA) analyzed to determine cfDNA fragmentation hotspots enriched in BCa patients. The hotspots were used as features in the machine learning models, which were contrasted with models using other cfDNA-derived features. Functional bioinformatics analyses were used to ascertain the biological significance of cfDNA hotspots in BCa pathogenesis. Both methods yielded similar results [110].

The results demonstrated that the cfDNA hotspot-based machine learning model achieved an area under the curve (AUC) of 0.96, with a sensitivity of 87% and a specificity of 100%, indicating that the model correctly identified 87% of BCa cases while eliminating false positives. This level of specificity is clinically pertinent, as it reduces the risk of overdiagnosis and unnecessary follow-up tests. Furthermore, stage-stratified analysis showed that for early-stage BCa (low-grade Ta and T1 tumors), the model sensitivity was 71% at 100% specificity, whereas for advanced BCa (high-grade T1 and MIBC), the sensitivity was 92% at 100% specificity. These findings suggest that cfDNA hotspot analysis is especially effective in detecting aggressive BCa cases, which is crucial for early intervention and treatment planning.

Bioinformatics analyses demonstrated that cfDNA hotspots captured BCa-specific molecular features, including regulatory elements, transcription factor binding sites, chromosome loci previously linked to BCa risk in genome-wide association studies, and somatic mutations frequently found in BCa tumors. This means that hotspots for cfDNA are not random but meaningful biologically active markers for bladder cancer and depict the underlying molecular pathology of cancer.

Despite these promising findings, certain hurdles need to be bridged before incorporating cfDNA- and ctDNA-based tests into daily clinical practice. First, rigorous validation studies need to be conducted to establish that these results can be replicated in other groups to show consistency and therapeutic benefits. Second, because decreased sensitivity is observed when early malignant disease is present (71%), false negatives may be a cause for concern, which may compromise early detection. Biological processes, such as tumor shedding variability, breakdown of cfDNA, and urine sampling protocols, may also be responsible for such limitations and therefore need to be explored [111].

Moreover, head-to-head correlations with established mature urinary biomarkers, such as UroVysion (FISH), CxBladder, NMP22, and Bladder EpiCheck, are needed to determine whether cfDNA-based methods are better than existing tests. Although cfDNA fragmentomics and ctDNA mutation analysis provide molecular information that is not possible with conventional biomarkers, whether they will be cost-effective and practical for application in everyday clinical practice is yet to be seen. Standardization of sample handling procedures, depth of sequencing, and analysis is necessary for regulatory approval and clinical utility [112].

ctDNA and cfDNA fragmentomics are noninvasive frontiers for BCa diagnosis with high accuracy in disease detection and disease progression monitoring. The cfDNA hotspot-based machine learning model had excellent specificity (100%) and very good sensitivity (87%), with even greater sensitivity for advanced-stage BCa (92% sensitivity). Furthermore, ctDNA analysis yields actionable mutation detection to guide targeted therapy and personalized medical approaches. However, before it can be applied to substitute or complement traditional diagnostic devices, further multicenter validation, cost-effectiveness analysis, and head-to-head comparisons with existing urinary biomarkers are required. Future research should focus on optimizing cfDNA and ctDNA detection strategies, improving early stage sensitivity, and using AI-aided data interpretation to enhance predictive models. If they are validated, these noninvasive methods could transform BCa diagnosis, reducing the requirement for invasive procedures while enabling earlier detection and tailored disease management [110].

## 7. Urinary Vesicles

Extracellular vesicles (EVs) are released by cells into bodily fluids and carry various biomolecules that represent the physiological conditions of their parental cells. Among them, urinary extracellular vesicles (uEVs) are also promising candidates as cancer biomarkers, offering a noninvasive and clinically applicable method for the detection of diseases. The aim of this study was to establish a uEV-derived mRNA signature that can act as a biomarker for early BCa diagnosis and prognostic evaluation [113].

To identify a potential mRNA signature, transcriptomic sequencing was performed on EVs isolated from a normal bladder cell line and multiple bladder cancer cell lines of varying grades. Three mRNAs, serglycin (*SRGN*), friend leukemia integration 1 transcription factor (*FLI1*), and *MACROH2A2*, were significantly altered in BCa cells compared with normal cells in this study. The diagnostic potential of these biomarkers was verified in a clinical urine sample set (n = 97) consisting of healthy controls, BCa patients, and post-surgical BCa patients. Quantitative RT–qPCR confirmed differential patterns of expression of these mRNAs that revealed the three-mRNA panel possessed extremely high diagnostic specificity with a very high area under the receiver operating characteristic curve (AUC = 0.973) for BCa diagnosis. The panel also efficiently distinguished between early stage BCa patients and normal controls (AUC = 0.969), evidencing its immense potential as an early diagnostic tool.

In addition to diagnosis, the present work also addressed the prognostic relevance of these mRNAs by determining their relative content in post-surgical urine. Notably, all uEV-mRNAs were significantly downregulated during surgery, indicating their value as response-to-therapy and disease-progression markers. These findings support the clinical significance of uEV-derived mRNA biomarkers, even more so with the availability and lack of invasiveness inherent to urine over traditional diagnostics by cystoscopy. With further validation in larger cohorts of patients, such uEV-mRNAs could be implemented into clinical applications as liquid biopsy biomarkers for BC early detection, recurrence tracking, and evaluation of response to therapy. The work here serves to illustrate the potential of using uEVs in precision oncology, and one avenue for better BCa care by noninvasive molecular diagnostics.

## 8. Metabolomics

Urinary metabolic biomarkers can enhance the precision and simplicity of BCa diagnosis by using liquid chromatography-mass spectrometry (LC-MS). Eighty urine samples were obtained from 50 patients with BCa (40 NMIBC and 10 MIBC) and 30 healthy controls. Metabolic profiling was performed using LC-MS to identify BCa-related differentially expressed metabolites, and binary logistic regression was used to construct biomarker panels that could distinguish BCa patients from normal individuals and NMIBC from MIBC. Correlation analysis and network modeling have also been used to examine the metabolic pathways involved in the pathogenesis of BCa [114]. The analysis identified 26 urinary metabolites that were different in BCa patients compared to healthy controls, reflecting distinct metabolic changes with BCa development. Moreover, eight metabolites were found to differentiate NMIBC from MIBC, implying their utility in tumor staging and risk stratification. Two panels of biomarkers were constructed to enhance the diagnostic precision. The first panel, urine urate, 4-androstene-3α, 17β-diol, and 3-indoxyl sulfate, performed well with an AUC of 0.983, which indicates high sensitivity and specificity in distinguishing BCa patients from healthy individuals. The second panel of L-octanoylcarnitine, γ-glutamylleucine, and heptanoylcarnitine accurately discriminated NMIBC from MIBC with a high AUC of 0.941, indicating good predictive accuracy for tumor staging. In addition to its diagnostic significance, this study also probed the biochemical pathways underlying BCa progression by establishing a compound reaction enzyme–gene network. This indicates that purine, androgen, and amino acid metabolism are crucial for BCa pathogenesis. Most importantly, urate, a significant biomarker identified in this study, is a purine metabolism end product that has been implicated in cancer cell proliferation and the regulation of oxidative stress. Similarly, identification of the steroid hormone derivative 4-androstene-3α, 17β-diol points toward a hormonal imbalance being involved in BC development. The identification of L-octanoylcarnitine and heptanoylcarnitine points toward fatty acid oxidation and mitochondrial metabolism derangements, which are typically altered in aggressive BC phenotypes. These results suggest that metabolite panels are superior to single biomarkers in terms of diagnostic precision and provide information on the pathogenesis of BC. Their higher AUC values demonstrate that they are feasible, noninvasive tools for the detection and staging of BCa, and that they have the potential to eliminate the need for repeated cystoscopy in surveillance and diagnosis. In this study, it was possible to identify and verify BCa metabolic biomarker panels with improved diagnostic performance. They not only serve as a noninvasive alternative to cystoscopy but also provide insight into the genetic basis of breast cancer tumor development. With further validation in large clinical populations, biomarkers can be integrated into routine clinical practice, revolutionizing early identification, risk assessment, and tailored treatment planning for patients with BCa.

## 9. Conclusions

Although considerable progress has been made in urinary biomarker studies for the detection, staging, and surveillance of bladder cancer, there are still important scientific and clinical challenges that need to be overcome. Although many studies have reported promising metabolic, protein, RNA, and DNA methylation markers, their implementation in daily clinical practice is hampered by poor reproducibility, lack of validation, and the absence of standardized methods. Most biomarkers have been tested in small, single-center cohorts with heterogeneous patient populations, which generates bias and limits generalizability. In addition, while many biomarkers demonstrate excellent diagnostic performance in stringently controlled research settings, whether they are applicable in the real world is uncertain because of the absence of standardization of pre-analytical and analytical methodologies. Different studies have employed different sample collection protocols, sequencing platforms, and statistical analyses, which generate divergent results and hinder clinical translation.

Another major challenge is the high degree of overlap between malignant and benign urinary profiles, which generates false-positive results, particularly in patients with inflammation, urinary tract infection, or hematuria of nonmalignant etiology. This is at the expense of specificity, and highlights the need for more refined stratification models using molecular, clinical, and imaging data. Although some biomarkers, such as UroVysion™ and Bladder EpiCheck^®^, have shown clinical promise, none have demonstrated sufficient sensitivity and specificity to fully replace cystoscopy, which, for all its invasiveness, cost, and associated patient discomfort, is still the gold standard. Recent advances in bladder cancer diagnosis have been directed toward the detection of false positives. One such promising tool is the development of multi-marker panels that use a combination of two or more urinary or molecular markers to increase diagnostic specificity and reduce misclassification. For instance, panels that contain proteins such as calreticulin, γ-synuclein, and soluble catechol-O-methyltransferase have shown better sensitivity and specificity than single-marker assays [115]. Likewise, a multimodal evaluation combining cytology, FISH, ImmunoCyt, and NMP22 tests has reported up to 98% sensitivity in the identification of bladder cancer, while significantly reducing false negatives for high-grade tumors [116]. Integration of multi-omics approaches (genomics, transcriptomics, proteomics, and metabolomics) could potentially enable the identification of stable, composite biomarkers with improved bladder cancer detection and classification accuracy. In addition, the development of liquid biopsy technologies, such as ctDNA and exosomal RNA, may improve early detection, treatment response monitoring, and prognostic evaluation. Future biomarker research must make a paradigm shift toward machine learning and AI-based algorithms, which have great potential for biomarker panel optimization, improvement of diagnostic sensitivity, and reduction of false positives. These models can be trained on large annotated datasets to recognize patterns between cancerous and inflammatory conditions and yield a strong diagnostic technique [117]. A recent study presented a multispectral 3D DNA system with a multimodal machine-learning program that probed five protein signatures in bladder cancer-derived urine extracellular vesicles. It significantly improved the accuracy of diagnosis (95% accuracy, 93% recall) by effectively reducing the number of benign false positives [118]. The use of multi-marker panels with computational analysis is highly promising for diminishing diagnostic errors due to non-malignant urinary pathology.

Several key steps must be taken for urinary biomarkers to become a clinical reality. First, the establishment of internationally agreed guidelines for sample collection, storage, and biomarker analysis is essential to minimize variability between studies. Second, multicenter large-scale prospective validation studies are required to validate the diagnostic and prognostic value of urinary biomarkers in a wide range of patient populations. Third, rather than attempting to replace cystoscopy altogether, urinary biomarkers must be integrated into risk-adaptive surveillance algorithms to reduce unnecessary invasive tests without compromising high diagnostic precision. Fourth, sound cost-effectiveness studies are required to determine the economic viability of urinary biomarkers in the clinical environment. Finally, promising biomarkers must undergo regulatory clearance and validation in real-life settings through longitudinal clinical trials to test their impact on patient outcomes.

Despite these challenges, urinary biomarkers hold transformative potential for BCa management, offering a noninvasive, accessible, and patient-friendly alternative to traditional diagnostic methods. However, they are constrained by scientific, logistic, and economic limitations for large-scale applications. Future research will need to focus on standardization, verification, and implementation in the clinical setting with AI and multi-omics technologies utilized for the design of highly specific, noninvasive diagnostics. With rigorous testing and cross-sectional collaboration, urinary biomarkers will ultimately become a component of precision oncology to reduce the need for intrusive diagnostics and significantly improve early diagnosis, risk stratification, and monitoring during BCa therapy.

## Figures and Tables

**Table 1:** Overview of the studies evaluating the performance of automated urine particle analyzers for the detection of atypical cells.

Automated Particle Analyzer	Patient Population	Reference Measure	Diagnostic Performance	Reference
iQ200 Analyzer	Patients with suspicious atypical cells	Urinary cytology and histopathology	Sensitivity: 87.5%	[28]
Sysmex UF-5000	Samples with >1 atypical cell/µL	Manual microscopy		[29]
Sysmex UF-5000	Specimens (163) from 128 patients	Urinary cytopathology	Sensitivity: 59.0% Specificity: 82.1% PPV: 75.0% NPV: 68.8%	[30]
Sysmex UF-5000	Patients (33) with any indication for a cystoscopy examination	Urine cytology on cystoscopy	Sensitivity: 27.0% Specificity: 78.0% PPV: 50.0% NPV: 56.0%	[31]
Sysmex UF-5000	Patients for which a urinary particle analysis was requested	Histological analysis	Sensitivity: 79.5% Specificity: 85.1%	[32]

Abbreviations: NPV, negative predictive value; PPV, positive predictive value.

## Abbreviations

The following abbreviations are used in this manuscript:

AI	Artificial Intelligence
ALOX5	Arachidonate 5-Lipoxygenase
APC	Adenomatous Polyposis Coli
AS1	Antisense RNA 1
AUC	Area Under the Curve
BCa	Bladder Cancer
BCD	Bladder Cancer Detection
BCG	Bacillus Calmette-Guérin
BE	Bladder EpiCheck
BLCAP	Bladder Cancer Associated Protein
BTA	Bladder Tumor Antigen
CA9	Carbonic Anhydrase 9
CD24	Cluster of Differentiation 24
CDK1	Cyclin-Dependent Kinase 1
CEP55	Centrosomal Protein 55
CI	Confidence Interval
CIS	Carcinoma In Situ
CRH	Corticotropin-Releasing Hormone
CT	Computed Tomography
CXCL16	C-X-C Motif Chemokine Ligand 16
CXCR2	C-X-C Motif Chemokine Receptor 2
CYFRA21	Cytokeratin Fragment 21-1
DNA	Deoxyribonucleic Acid
DOR	Diagnostic Odds Ratio
ELISA	Enzyme-Linked Immunosorbent Assay
EMT	Epithelial-Mesenchymal Transition
ERBB2	Erb-B2 Receptor Tyrosine Kinase 2
FDA	Food and Drug Administration
FGFR	Fibroblast Growth Factor Receptor
FISH	Fluorescence In Situ Hybridization
FLI1	Friend Leukemia Integration 1 Transcription Factor
GALR1	Galanin Receptor 1
HAND2	Heart And Neural Crest Derivatives Expressed 2
HG	High Grade
HGUC	High-Grade Urothelial Carcinoma
HOTAIR	HOX Transcript Antisense RNA
HOXA13	Homeobox A13
HPF	High Power Field
HR	Hazard Ratio
HRAS	Harvey Rat Sarcoma Viral Oncogene Homolog
IGFBP5	Insulin-Like Growth Factor Binding Protein 5
IQGAP3	IQ Motif Containing GTPase Activating Protein 3
KRAS	Kirsten Rat Sarcoma Viral Oncogene Homolog
LDA	Linear Discriminant Analysis
LGBC	Low Grade Bladder Cancer
MALAT1	Metastasis Associated Lung Adenocarcinoma Transcript 1
MCM5	Minichromosome Maintenance Complex Component 5
MDK	Midkine
MET	Mesenchymal-Epithelial Transition Factor
MIBC	Muscle-Invasive Bladder Cancer
MLL	Mixed Lineage Leukemia
MN	Micronucleus
MRI	Magnetic Resonance Imaging
MS	Mass Spectrometry
MSP	Methylation-Specific PCR
NB	Nuclear Budding
NMIBC	Non-Muscle-Invasive Bladder Cancer
NMP22	Nuclear Matrix Protein 22
NPV	Negative Predictive Value
NRN1	Neuritin 1
OF	Observed Frequency
ONECUT2	One Cut Homeobox 2
OTX1	Orthodenticle Homeobox 1
PCR	Polymerase Chain Reaction
PENK	Proenkephalin
PLR	Positive Likelihood Ratio
PLS	Partial Least Squares
POC	Point of Care
PPV	Positive Predictive Value
PTK2	Protein Tyrosine Kinase 2
RNA	Ribonucleic Acid
ROC	Receiver Operating Characteristic
RP5	Reference Sequence 5
RT	Reverse Transcription
SERS	Surface-Enhanced Raman Scattering
SOC	Standard of Care
SRGN	Serglycin
TCGA	The Cancer Genome Atlas
TERT	Telomerase Reverse Transcriptase
